# Formation of a structurally-stable conformation by the intrinsically disordered MYC:TRRAP complex

**DOI:** 10.1371/journal.pone.0225784

**Published:** 2019-12-02

**Authors:** Edmond J. Feris, John W. Hinds, Michael D. Cole

**Affiliations:** 1 Department of Molecular and Systems Biology, Geisel School of Medicine at Dartmouth College, Hanover, NH, United States of America; 2 Norris Cotton Cancer Center, Dartmouth-Hitchcock Medical Center, Lebanon, NH, United States of America; University of Colorado Denver Skaggs School of Pharmacy and Pharmaceutical Sciences, UNITED STATES

## Abstract

Our primary goal is to therapeutically target the oncogenic transcription factor MYC to stop tumor growth and cancer progression. Here, we report aspects of the biophysical states of the MYC protein and its interaction with one of the best-characterized MYC cofactors, TRansactivation/tRansformation-domain Associated Protein (TRRAP). The MYC:TRRAP interaction is critical for MYC function in promoting cancer. The interaction between MYC and TRRAP occurs at a precise region in the MYC protein, called MYC Homology Box 2 (MB2), which is central to the MYC transactivation domain (TAD). Although the MYC TAD is inherently disordered, this report suggests that MB2 may acquire a defined structure when complexed with TRRAP which could be exploited for the investigation of inhibitors of MYC function by preventing this protein-protein interaction (PPI). The MYC TAD, and in particular the MB2 motif, is unique and invariant in evolution, suggesting that MB2 is an ideal site for inhibiting MYC function.

## Introduction

Cancer cells evolve through a multistage process, driven by the progressive accumulation of multiple genetic and epigenetic abnormalities. Despite the complexity of carcinogenesis, the process is fragile: the growth and survival of cancerous cells can be impaired by the inactivation of a single oncogene [1]. Altered transcriptional programs can also make cancer cells highly dependent on certain regulators of gene expression [2]. Therefore, research into mechanisms of cellular proliferation carries the promise of discovering new therapies. Extensive studies of tumor genomes have revealed recurrent somatic mutations that affect normal transcriptional control [2]. One of these is MYC, a master regulator of transcription. MYC plays a central role in carcinogenesis and is the most wanted target for drugs that perturb dysregulated transcriptional programs. The fact that many cancer cells cannot survive without MYC–a phenomenon termed “MYC addiction”–provides a compelling case for the development of MYC-specific targeted therapies.

Deregulated expression of MYC is a hallmark of 70% of all cancers [3] and *MYC* is the most frequently amplified gene in human cancer. Furthermore, a diverse array of mutations in oncogenic signaling pathways can lead to MYC overexpression [4,5]. Relatively small changes in MYC protein levels can promote or block oncogenic transformation or cancer development. The biological functions of MYC may be broader than of any other gene. These functions include controlling proliferation, promoting oncogenic transformation, inducing tumor formation, blocking differentiation, inducing apoptosis, inducing G2 arrest, and altering the inherited predisposition to cancer, among others [6].

The MYC family has three members: c-MYC (MYC), N-MYC (or MYCN) and L-MYC (or MYCL). During the life of an organism, MYC is universally expressed in all proliferating cells, whereas MYCN is often co-expressed with MYC in stem cells and other primitive lineages [7,8]. While MYCN is amplified in a subset of tumors, MYC is the most frequently deregulated gene in cancer [9,10]. Although all three members of the MYC family differ in cellular expression and chromosomal locus, their protein products consist primarily of the same two domains: an N-terminal transactivation domain and a C-terminal DNA binding domain. The C-termini of all MYC family proteins are highly conserved and include a basic helix-loop-helix/leucine zipper (bHLH/LZ) motif, and the basic region is required for sequence-specific interaction with DNA [11,12]. The N-termini of MYC family members have four main regions of conservation. These regions were designated as MYC homology boxes 1 through 4 (MB1-4) [13]. The MBs, especially MB2, are evolutionarily conserved, extending from humans to sponge [14]. MB2 is necessary for both transactivation and repression of MYC’s ‘classical’ target genes [15–17].

Since MYC has no inherent enzymatic activity, it is sometimes thought to be “undruggable” [18,19]. However, there is hope that PPIs involving MYC can be targeted therapeutically. The MYC DNA-binding domain heterodimerizes with MAX and together they form a tight complex with DNA. Several labs have attempted to find small molecules that inhibit the MYC:MAX interaction with limited success [18–20]. One difficulty in targeting this protein-protein interface is that it involves extensive contacts throughout the helix-loop-helix and leucine zipper domains, and countless other transcription factors share these motifs [11]. Hence, it is very difficult to inhibit MYC:MAX heterodimers without also introducing off-target side effects on other HLH, LZ, or coiled-coil proteins.

The MYC TAD is also involved in several PPIs, including an interaction with the TRansformation/tRanscription domain-Associated Protein (TRRAP). TRRAP has been shown to be a critical MYC cofactor [21–23], and MB2 is required for MYC:TRRAP binding [23,24]. TRRAP is a member of various histone-acetylation (HAT) complexes which aid transcription factors, like MYC, in controlling gene expression. The identification of TRRAP as an essential MYC cofactor established a link to HAT complexes containing GCN5 and TIP60 and provided an important mechanistic insight into MYC’s function [17,19,22,23,25]. TRRAP is a highly conserved 434 KDa protein that belongs to the Phosphoinositide 3-Kinase-related kinase (PIKK) family that includes mTOR, DNA-PKcs, ATM/Tel1, ATR/Mec1 and SMG-1 [26,27]. PIKKs are kinases involved in transcriptional regulation, DNA repair, cell growth, metabolic control and mRNA surveillance, but TRRAP lacks a kinase domain and has no enzymatic activity throughout evolution [22,28]. Instead, TRRAP is thought to function as a scaffold, bridging transcription factors and chromatin modifying complexes [29,30]. *TRRAP* is an essential gene, and its disruption leads to early embryonic lethality in mice [25,31]. TRRAP mutations have been associated with tumorigenesis, and some models portray TRRAP as an oncogene [32,33]. It is difficult to reconcile the proposed function of TRRAP as a mere scaffold and the observations regarding its role in the cell cycle and disease. Further investigation into the biological functions of TRRAP is warranted.

TRRAP is massive and is involved in various megadalton sized protein complexes. It is a subunit of both the STAGA and NuA4 HAT complexes, which contain GCN5 and Tip60 respectively [19,25,34]. Although it lacks catalytic activity, TRRAP is critical for transcriptional coactivator function and enables the activities of STAGA and NuA4 to be directed at specific genes in order to stimulate their expression [35]. These complexes use TRRAP to mediate their interactions with transcription factors, like MYC, E2F, E1A and p53, making it a conserved activator target in all eukaryotes [23,36,37]. TRRAP recruitment to DNA results in transcriptional activation by enabling histone modification around gene promoters and hyperacetylation of lysine residues on histone tails [22,38].

The MYC:TRRAP interaction has been roughly mapped [23,39], however, the precise domains of PPI have not been described. McMahon et al. established that MB2 is required for the MYC:TRRAP interaction but did not describe the minimal MYC domain that is sufficient for TRRAP binding. Similarly, the minimal sufficient MYC-binding domain of TRRAP has not been described [39]. The identification of these minimal domains required for the MYC:TRRAP PPI is important for further studies.

More recently, a Cryo-EM structure of *Saccharomyces cerevisiae* Tra1p was reported to 3.7 Å resolution revealing the extensive network of α-helical solenoids [40]. An atomic model was built with 3474 residues assigned with visible side-chains, but 270 residues were not resolved in the reconstruction. These unresolved residues were distributed across chain breaks that contain either loops or disordered regions. Tra1p was found to have HEAT, FAT, FRB, kinase and FATC domains arranged sequentially from N- to C- terminus, which are characteristic of PIKK family proteins [26,27]. A prediction of TRRAP’s secondary structure, aligned with Tra1p, revealed 98% overlap in helical repeats, even though the sequences of the two proteins are only 27% identical [41].

Here we report further characterization of the MYC:TRRAP interaction. We describe the minimal binding domains for MYC:TRRAP, their critical regions of interaction, the secondary structure of the domains, and the induction of a stable MYC:TRRAP complex using chemical chaperones. These studies should form a basis for developing small molecule inhibitors of the MYC:TRRAP interaction.

## Materials and methods

### Cell culture

HEK293T cells from ATCC® (CRL-3216^™^) were maintained in DMEM supplemented with 10% fetal bovine serum and prophylactic Plasmocin^™^ (InvivoGen) to prevent mycoplasma contamination. The HEK293T cell line is a highly transfectable derivative of human embryonic kidney 293 cells and contains the SV40 T-antigen. LookOut® Mycoplasma PCR Detection Kit (Millipore Sigma) was used to check for mycoplasma contamination every six months.

### Deletion mapping co-immunoprecipitation

The indicated TRRAP constructs were cloned into a CMV-driven plasmid containing an N-terminal FLAG tag as previously described [23]. Full-length wild-type (WT) MYC and MYC ΔMB2 (Δ129–145), and the indicated MYC constructs were cloned into the same CMV-driven plasmid but containing a Glu-Glu (PYO) tag instead. HEK293T cells were co-transfected with equal amounts of each plasmid using LipoD293^™^ In Vitro DNA Transfection Reagent per protocol (SignaGen). Cells were plated subconfluently 16–20 hours prior to transfection. After 24 hours, cells were lysed in F-buffer (10 mM TRIS pH 7.5, 50 mM NaCl, 30 mM sodium pyrophosphate, 5 mM ZnCl_2_, 10% glycerol, 1% Triton-X, 50 mM NaF) supplemented with 1 mM PMSF, 10 μM Leupeptin, 10 μM Pepstatin-A, and 10 μg/mL Aprotinin for immunoprecipitations and co-immunoprecipitations. Immunoprecipitations were performed using anti-FLAG (Sigma Aldrich), anti-PYO (Covance), or anti-MYC (C33 Santa Cruz Biotechnology) agarose pre-conjugated beads. Co-immunoprecipitation was analyzed by western blots with the following antibodies: MYC (sc-764 Santa Cruz Biotechnology), TRRAP (A301-132A Bethel Laboratories), FLAG (F7425 Sigma Aldrich), and PYO (Covance).

### Protein production and purification

The indicted MYC or TRRAP constructs, or MYC-TRRAP fusions were cloned into a modified pGEX 6P-1 vector containing an additional C-terminal TwinStrep tag II (TS) from IBA Life Sciences. BL21 (De3) *E*. *coli* were transformed with each of these vectors and stored at -80°C in a 25% glycerol solution. A starter culture was prepared by adding a small amount of glycerol stock to 25 mL Terrific Broth (TB; BD Biosciences) with 50 ug/mL ampicillin. Next, this culture was incubated in a shaker overnight at 37°C/250 RPM and then divided into five 2 L flasks containing 500 mL of TB supplemented with ampicillin. After the OD of the culture reached 2.0, the flasks were placed in an ice bath until the temperature of the culture reached 16°C. Finally, Isopropyl β-D-1-thiogalactopyranoside (IPTG) was added to a final concentration of 1 mM and the culture was incubated in a shaker at 16°C/250 RPM for 20–24 hours. The culture was subsequently centrifuged at 4°C, 6,000 RCF for 20 minutes and the pellet stored at -80°C until purification.

The frozen pellets were resuspended for lysis in 250 mL of a solubility-optimized buffer for MYC constructs containing: 100 mM TRIS, 150 mM NaCl, 5% Ethylene Glycol (EG), 1mM EDTA, 1mM TCEP, and 0.02% NaN_3_. Additionally, lysozyme was added at 1 mg/mL and protease inhibitors including: 1 mM PMSF, 10 μM Leupeptin, 10 μM Pepstatin A, and 10 μg/mL Aprotinin. The lysate was kept on ice for 30 min, sonicated at 70% amplitude with a Branson 250 sonicator for 3 min (10 sec ON, 10 sec OFF), and spun >100,000 RCF for 60 min. The lysate was collected, and the pellet discarded. Using an NGC chromatography system (Bio-Rad), a 5 mL GSTrap (GE Healthcare) affinity column was used to purify the indicated GST-fusion construct from the lysate. Following elution with the same lysis buffer minus lysozyme and protease inhibitors but supplemented with 20 mM reduced glutathione, the eluate was incubated overnight in the presence of HRV-3C protease (ThermoFisher) for the removal of the GST tag. Then, the products of this reaction were loaded onto a 5 mL StrepTactin XT ® column (IBA Life Sciences) using the same chromatography system. After washing with the same buffer as above, constructs were eluted with 50 mM Biotin. This eluate was then incubated with Ac-TEV protease (ThermoFisher) for the removal of the TS tag. The products of this reaction were passed through a Ni-NTA gravity column (QIAGEN) for the removal of the Ac-TEV protease. The flow-through was concentrated and loaded on to a SEC Superdex 200 16/600 column (GE Healthcare) previously equilibrated with 1X PBS. Following elution, purity was confirmed using SDS-PAGE (S2 Fig). Protein concentration was quantified using spectrometric analysis, and aliquots were flash-frozen in liquid N_2_ and stored at -80°C.

^15^N-labeled proteins were purified exactly as above. However, expression in *E*. *coli* differed. Starters were added to 250 mL of TB; the culture was incubated until an OD of 4.0 was reached. Then, the bacteria were centrifuged at 500 RCF for 20 min to remove the TB media. The pellet was then resuspended in minimal media (M9 media) containing 0.75 g ^15^NH_4_Cl and unlabeled dextrose. The culture was then incubated with 1 mM IPTG for protein induction and harvested as outlined above.

### Circular dichroism spectroscopy

The secondary structure of the indicated protein constructs (1 μM) was measured in 1X PBS with or without the indicated additives. CD spectra were acquired from 200 to 250 nm at 25°C in a Jasco J-185 instrument using a 10 mm spectrosil cuvette (VWR). The mean residue ellipticity (MRE) was calculated using the equation:

$$[\theta ]=\theta \frac{M}{10LC}$$ (1)

where *[θ]* is the MRE, *θ* is the measured ellipticity in millidegrees, *M* is the average molecular weight in g/mol, *L* is the path length of the sample cell in centimeters, and *C* is the concentration of the protein in g/L.

### *In vitro* pulldown

The specified purified MYC and C-terminal TS-tagged TRRAP protein constructs were mixed at 50 μM each and incubated at room temperature for 2h in the presence of StrepTactin XT beads in 1X PBS. After pulldown, bound proteins were eluted with 50 mM Biotin and analyzed with a Coomassie-stained SDS-PAGE. For MYC-TRRAP fusion proteins, the specified constructs were incubated with and without 30% ethylene glycol (EG) in 1X PBS for 30 min before linker cleavage with HRV-3C. Afterwards, the same pulldown and analysis followed.

### Size-exclusion chromatography in ethylene glycol

A Superdex 200 16/600 column connected to an NGC chromatography system was used like above. The column was first equilibrated in 1X PBS supplemented with 30% EG. The indicated protein constructs were loaded onto the column and λ280 spectra were collected in real-time. Due to a high system pressure, the flow rate for this method had to be reduced to 0.5 mL/min.

### NMR spectroscopy

Both ^1^H measurements and ^1^H,^15^N-HSQC measurements were recorded at 25°C with a 500 MHz Bruker NMR spectrometer equipped with a standard probe using 3 mm sample tubes. Unlabeled MYC 120–161 ^1^H spectra were recorded in either 1X PBS or with 30% TFE-d_2_. ^1^H,^15^N-HSQC spectra of MYC 120–161 and MYC 120-161-TRRAP 2033–2088 were recorded in 1X PBS with 30% TFE-d_2_. Data was processed using TopSpin 4.0 (Bruker) and visualized using NMRFAM-SPARKY software [42].

### Statistics

All experiments were repeated at least 3 times. An unpaired student’s t-test was performed to determine standard deviation and statistical significance. P-value ≤ 0.05 was considered statistically significant. Error bars represent SEM.

## Results

### Mapping the MYC:TRRAP interaction

**Fig 1:**
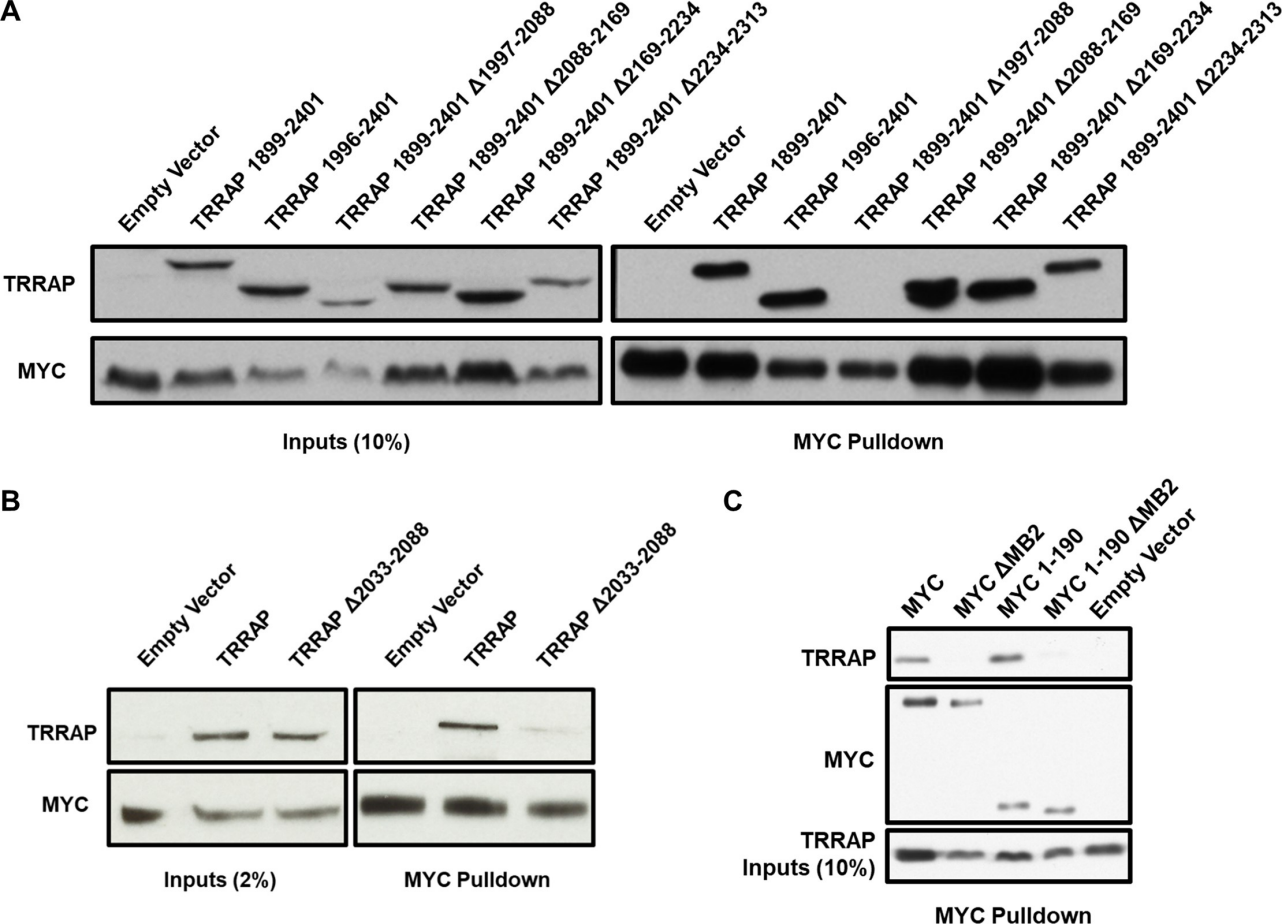
Minimal interacting domains of MYC:TRRAP. (A) The indicated regions of TRRAP were cloned into a CMV-FLAG expression vector. Proteins were co-expressed with PYO-tagged full-length MYC (1–439) and then MYC was IPed with anti-PYO beads. Co-IP was assessed by western blot with anti-FLAG. The most critical binding domain is within residues 1997–2088. (B) Full-length TRRAP (1–3830) and TRRAP Δ2033–2088 were cloned into a CMV-FLAG expression vector and transfected into HEK293T cells. Proteins were co-expressed with PYO-tagged full-length MYC and then MYC was IPed with anti-PYO beads. Co-IP was evaluated by western blot. TRRAP Δ2033–2088 shows reduced binding to MYC. (C) Full-length MYC, MYC ΔMB2, MYC 1–190, and MYC 1–190 ΔMB2 were cloned into a CMV-PYO expression vector and transfected into HEK293T cells. Proteins were co-expressed with FLAG-tagged TRRAP 2033–2283 then MYC was IPed with anti-PYO beads. Co-IP was evaluated by western blot. TRRAP 2033–2288 shows equal co-IP with full-length MYC as with MYC 1–190, and both require MB2.

Mapping of the MYC:TRRAP interaction was initiated with a series of external and internal deletions within residues 1899–2401 of TRRAP [39]. These deletions were constructed using proline residues as boundaries which largely correspond to the HEAT repeat boundaries [41]. Through a series of co-immunoprecipitation experiments, the most critical MYC-interacting region in TRRAP was determined to be within residues 1997–2088, without which the TRRAP:MYC interaction cannot occur in transient assays (Fig 1A). Although transient expression of the MYC protein and these TRRAP constructs vary significantly, it is still clear that the construct that lacks residues 1997–2088 of TRRAP is the most defective MYC binder. These TRRAP constructs were aligned with the results described by Knutson and Hahn and Díaz-Santín et al. [40,41]. Structural predictions for the most critical region suggest that it is inherently disordered, unlike its flanking domains.

To validate the mapping data above, a similar domain dependence was studied with full-length proteins. An expression construct for full length TRRAP (1–3830) was created, plus a similar construct lacking only the predicted intrinsically disordered domain (amino acids 2033–2088). Thus, the latter lacks only 55 amino acids out of the native 3830 amino acids in TRRAP. Fig 1B shows this small deletion mutant is defective for interaction with full length MYC. In this experiment the transient expression is consistent for each construct used. Thus, the intrinsically disordered region in TRRAP (2033–2088) is necessary for MYC interaction. The identification of a clear region that is necessary for the MYC:TRRAP interaction in human cells is inconsistent with the conclusions drawn from previously published mapping studies [39]. The data presented here suggests that solubility and/or conformational differences exist within this region of TRRAP when produced in bacteria, which can be the cause of the inconsistency between these two mapping studies.

Similar mapping studies were conducted on MYC (1–439) to define its domain of interaction with TRRAP. Stable binding appears to require amino acids 1–190 of MYC, which encompass a large portion of the TAD. Importantly, an internal deletion of MB2 (17 amino acids) within this domain largely eliminates TRRAP binding, consistent with earlier studies (Fig 1C) [23]. The relatively large domains in both TRRAP and MYC required for a stable interaction may suggest an extended protein-protein interface, although large domains may also be required to ensure proper folding of a small protein-protein interface. The fact that small deletions within each domain can eliminate binding (i.e., TRRAP 2033–2088 and MYC MB2) indicates these small regions may be the most crucial sites for binding and, therefore, small molecule targeting.

To validate the MYC’s minimal domain of interaction (i.e., MYC 1–190), a co-IP experiment was performed, testing the binding of this domain to endogenous TRRAP. MYC 1–190 co-IPs with endogenous TRRAP, and this interaction requires MB2 (S1A Fig). Finally, another co-IP experiment was performed to test the importance of the MB2 W135 amino acid (S1B Fig) which is essential for MYC-driven cellular transformation [21,23]. W135 of MYC is indispensable for the co-IP of the complex.

### Secondary structure of MYC and TRRAP

To gain further insight into the secondary structure of MYC:TRRAP, we produced pure protein constructs in large quantities in *E*. *coli* (S2A Fig). This involved sequential affinity purification using a GST N-terminal tag and a C-terminal TS tag. Upon tag removal, size-exclusion chromatography (SEC) was used to assess the monomeric state of protein constructs and to buffer exchange. This resulted in very pure and highly concentrated protein constructs meeting the requirements for structural determination experiments (S2B Fig).

**Fig 2:**
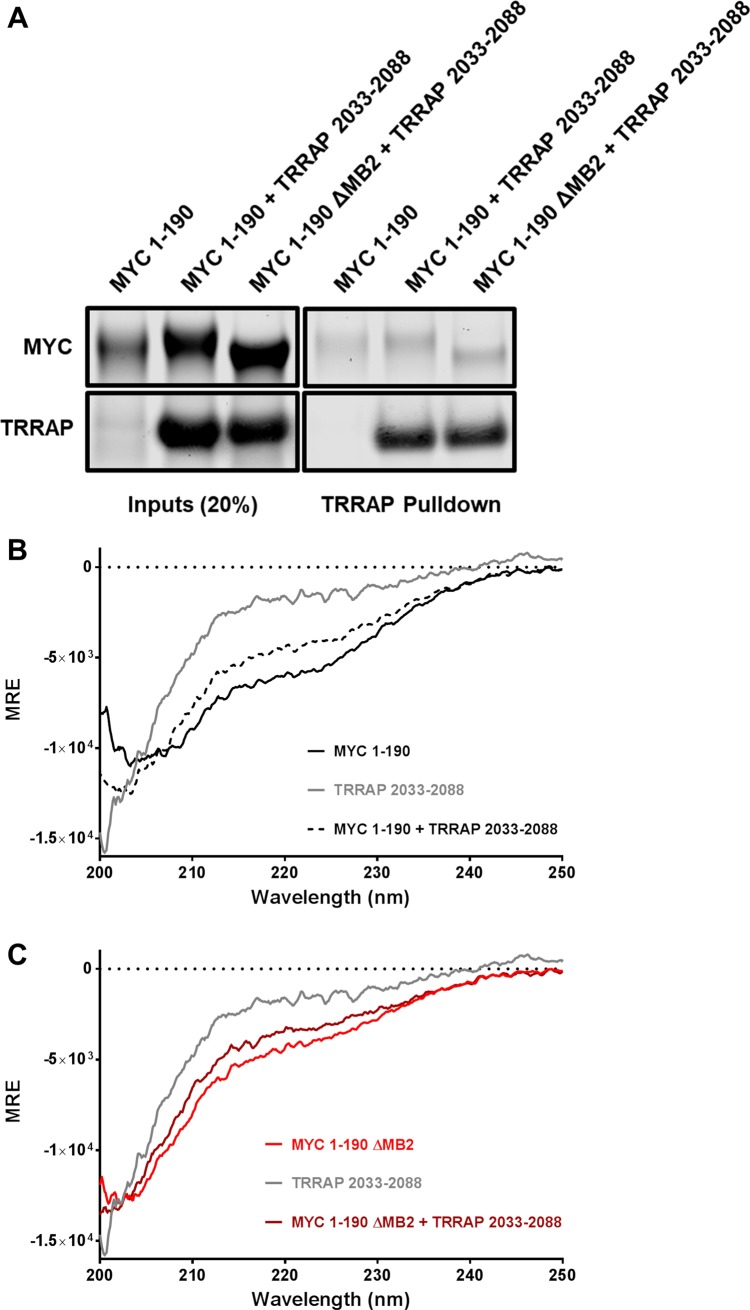
A MYC:TRRAP complex does not form *in vitro*. (A) Coomassie-stained SDS-PAGE of a TS tag pulldown of TRRAP 2033–2088 mixed with MYC 1–190 and MYC 1–190 ΔMB2 at 50 μM each. This result demonstrates that MYC 1–190 and TRRAP 2033–2088 do not interact when mixed *in vitro*. (B-C) CD spectra of MYC 1–190 and MYC 1–190 ΔMB2 mixed *in vitro* with TRRAP 2033–2088 at 10 μM each. CD spectra show no gain in secondary structure after mixing either MYC 1–190 or MYC 1–190 ΔMB2 with TRRAP 2033–2088.

The secondary structures of the MYC TAD and TRRAP 2033–2088 were evaluated by CD spectroscopy (Fig 2B). Although TRRAP 2033–2088 was suspected to be intrinsically disordered, nothing of its actual structural conformation was known [41]. CD measurements revealed that this domain of TRRAP is, in fact, an IDR, lacking any measurable α-helical or β-sheet secondary structure. The MYC TAD has also been described as an IDR, but the largest domain ever studied was MYC 1–143 [43]. CD spectra of MYC 1–190 confirms that the MYC TAD is largely disordered but with some helical characteristics, consistent with previous findings [43,44]. However, the deletion of MB2 removes the minimum at 222 nm while conserving the minimum at 208 nm (Fig 2C). This suggests that MB2 contains some of the α-helical elements attributed to the MYC TAD. With the hypothesis that MYC and TRRAP acquire a stable conformation upon binding, CD was used to test whether there was any gain in newly acquired secondary structure upon mixing MYC 1–190 and TRRAP 2033–2088. Fig 2 shows that there was no gain in secondary structure when these two constructs were mixed *in vitro* at a 1:1 molar ratio. There was no change in measurements at concentrations between 1–10 μM. Finally, a co-IP experiment was performed with purified proteins to determine whether MYC 1–190 and MYC 1–190 ΔMB2 exhibited any difference in binding to TRRAP 2033–2088. The proteins were mixed at a 1:1 molar ratio (50 μM each). Binding was assayed by Coomassie-stained SDS-PAGE (Fig 2A). There was no difference of TRRAP 2033–2088 binding to either MYC construct, suggesting that MYC 1–190 does not form a complex *in vitro*.

### Induction of an ordered structure on MYC:TRRAP

We explored alternative conditions to aid the formation of a protein complex *in vitro*. Although the regions of interaction of both MYC and TRRAP are IDRs, an ordered conformation could occur upon dimerization. Different methods of stabilizing an interaction have been described in the literature. Two precedents are the MYC:MAX crystal structure and the more recent NMR structure of the p53 TAD, both of which created protein-protein complexes from primary fusion constructs [11,45]. Furthermore, we tested additives or molecular chaperones that could induce secondary structure in MYC, TRRAP, and/or a MYC:TRRAP complex. To test different molecular chaperones, the secondary structure of each construct was characterized by CD spectroscopy in the presence of additives. These constructs included: MYC 1–190, TRRAP 2033–2088, and MYC 1–190 mixed *in vitro* with TRRAP 2033–2088 (S3 Fig). The additives tested included mostly osmolytes, along with some metal ions and organic solvents. Table 1 summarizes the results from these measurements.

**Table 1:** The effect of additives on MYC:TRRAP.

Additive	Secondary Structure
PBS	Unstructured
Glycerol	Partially α-helical
**Ethylene Glycol**	**α-helical**
Trehalose	Unstructured
Glycine	Unstructured
Betaine	Unstructured
Trimethylamine N-oxide	Unstructured
PEG400	Partially α-helical
PEG1500	Unstructured
PEG3350	Unstructured
PEG4000	Unstructured
PEG6000	Unstructured
PEG8000	Unstructured
PEG10000	Unstructured
PEG MME 2000	Unstructured
PEG MME 5000	Unstructured
ZiCl_2_	Unstructured
CuSO_4_	Unstructured
**2,2,2-Trifluoroethanol**	**Highly α-helical**
Methanol	Unstructured
Ethanol	Unstructured

**Fig 3:**
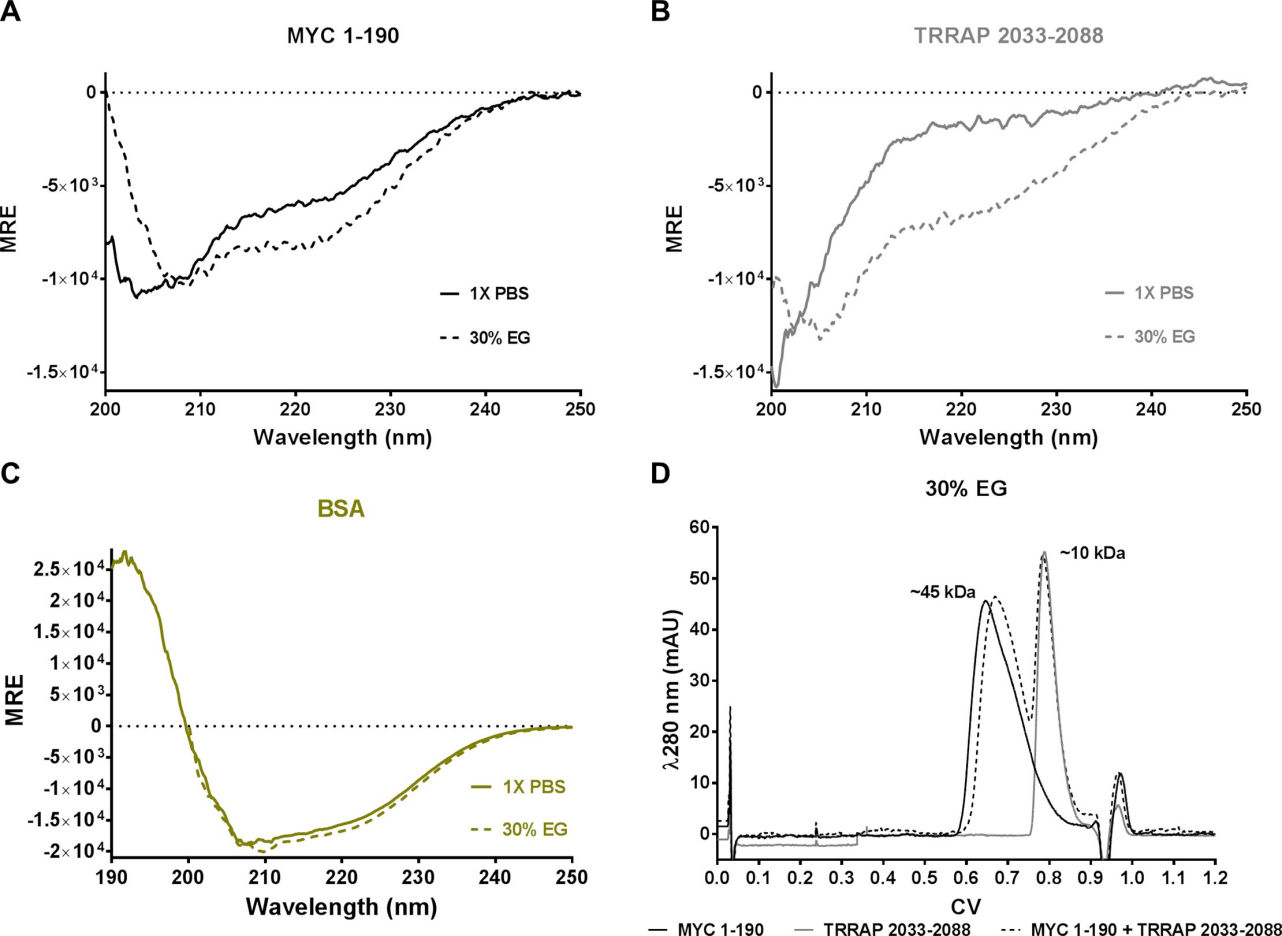
Effects of ethylene glycol on MYC and TRRAP. (A-C) CD spectra of MYC 1–190, TRRAP 2033–2088, and BSA. Solid lines represent measurements taken in 1X PBS; dotted lines represent measurements taken in 30% EG. A significant increase in the α-helical character of MYC and (to a lesser extent) TRRAP is observed in the presence of EG. However, BSA (a highly α-helical well-folded protein) appears unaffected by the presence of EG. D) SEC λ280 spectra of MYC 1–190 (black), TRRAP 2033–2088 (grey), and MYC 1–190 mixed with TRRAP 2033–2088 (black dotted) in 30% EG all at 100 μM. Neither MYC nor TRRAP showed any variation in their expected hydrodynamic radius as measured in 1X PBS. The mixed sample did not have any measurable tertiary peak that would indicate an association between the MYC and TRRAP.

Of the additives tested, ethylene glycol (EG) and 2,2,2-Trifluoroethanol (TFE) produced the most specific effect and the largest gain in secondary structure, respectively. EG induces a secondary structural change in both MYC and TRRAP, but not BSA (Fig 3A–3C). To test whether EG could induce a MYC:TRRAP complex, samples containing MYC 1–190, TRRAP 2033–2088, and MYC 1–190 mixed with TRRAP 2033–2088 (100 μM each) in 30% EG were run on an SEC column equilibrated with 30% EG (Fig 3D). Only two peaks were observed using the mixed sample, confirming that EG does not induce a MYC:TRRAP complex.

**Fig 4:**
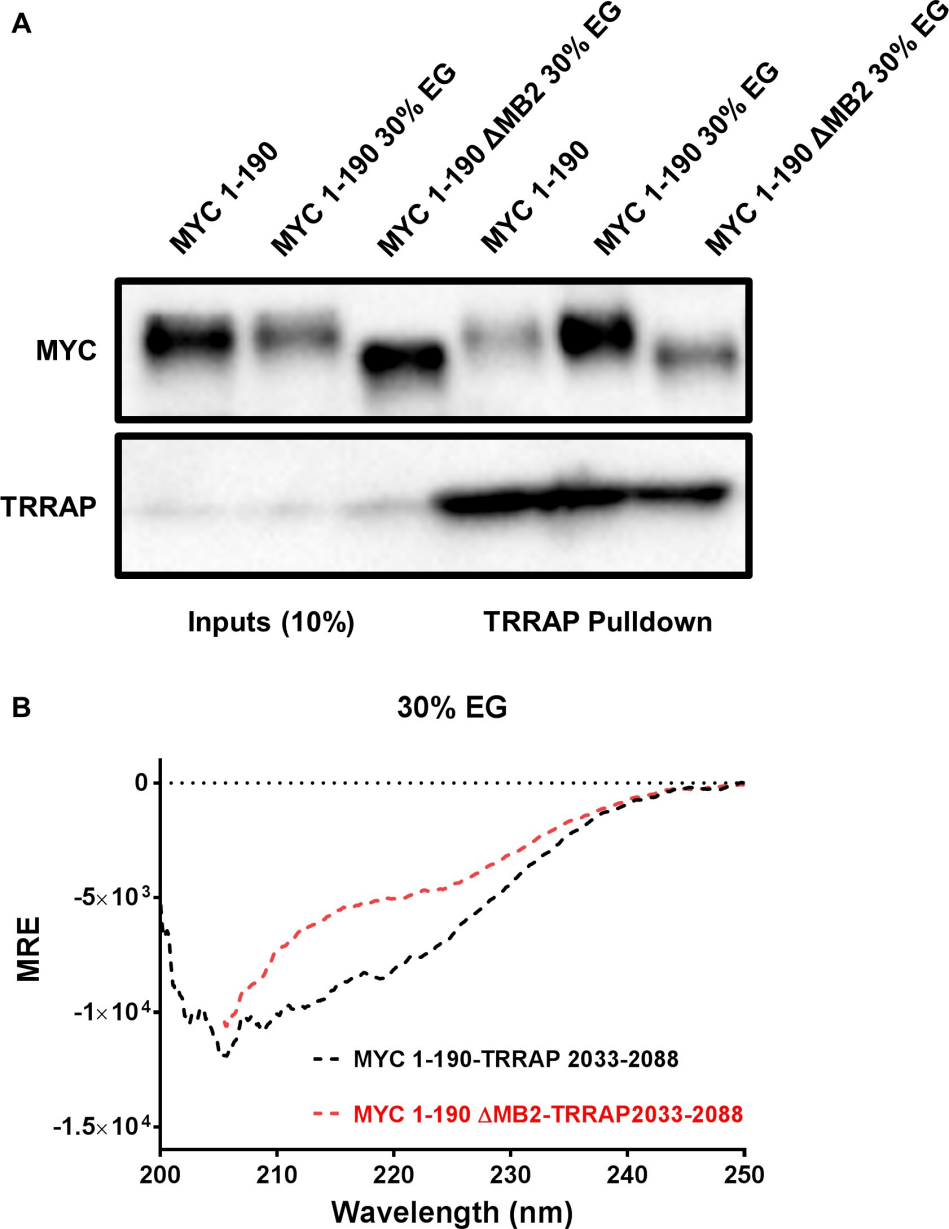
The effect of EG on MYC-TRRAP. (A) Coomassie-stained SDS-PAGE of a pulldown of two 3C protease-cleavable fusion proteins. MYC 1-190-TRRAP 2033-2088-TS and MYC 1–190 ΔMB2-TRRAP2033-2088-TS incubated in either 1X PBS or 30% EG. After 3C cleavage of the linker, the TRRAP domain was pulled down with StrepTactin® beads and the EG was washed away with 1X PBS. MYC 1–190 showed enhanced binding to TRRAP 2033–2088 in the presence of 30% EG but not in PBS, when compared to MYC 1–190 ΔMB2 in 30% EG. (B) CD spectra of two MYC-TRRAP fusion proteins in 30% EG: MYC 1-190-TRRAP 2033–2088 in black and MYC 1–190 ΔMB2-TRRAP 2033–2088 in red. The effects of EG on the fusion protein containing MB2 are more profound and are indicative of a specific gain in α-helical character.

The MYC-MAX and p53-CBP structures suggest that complexes of two IDRs can be established using a covalent linker. Therefore, expression of the minimal-interacting regions of TRRAP and MYC were produced as a fusion protein separated by a computationally-designed flexible linker (GSGSAGSAAGSGEFG) [46]. The effects of EG on a MYC-TRRAP fusion protein were compared to MYC ΔMB2-TRRAP using CD spectroscopy (Fig 4B). EG had a more profound effect on the secondary structure of the fusion protein containing MB2. This indicates that a fusion protein may be required to form a stable MB2-dependent MYC:TRRAP complex *in vitro*. To test whether a complex is being formed, two fusion proteins were produced with a cleavable 3C protease site between the MYC and TRRAP domains, MYC 1-190-TRRAP 2033-2088-TS and MYC 1–190 ΔMB2-TRRAP2033-2088-TS. After the addition of EG, the linker was cleaved, and any potential complex assessed by a pulldown experiment followed by Coomassie-stained SDS-PAGE (Fig 4A). These results suggest that a MYC:TRRAP complex was formed only in the presence of EG and that the complex remained bound after the cleavage of the linker and the removal of EG. Furthermore, the complex requires MB2, consistent with the complex that forms *in vivo*. These results point to a native-like complex, formed *in vitro* with the aid of a flexible linker and stabilizing additives.

### ^1^H, ^15^N-HSQC spectrum of MYC vs MYC-TRRAP

Because W135 of MYC is critical for cellular transformation and for the MYC:TRRAP interaction, an ^1^H, ^15^N-HSQC of MYC with W135 assigned would be extremely useful for screening inhibitors of MYC activity in cancer. The indole N-H pair in a tryptophan side-chain gives its chemical shift peak in the HSQC spectra a unique and distinctive appearance. Therefore, an HSQC spectrum of MYC could have the W135 side-chain N-H pair assigned without necessarily assigning all other peaks.

Since TFE induced the highest gain in secondary structure in MYC measured by CD (Table 1), NMR experiments were carried out to characterize the structural elements of MYC 120–161 and MYC 120-161-TRRAP 2033–2088 in the presence of TFE-d_2_. These constructs were chosen because W135 of MYC is the only tryptophan residue within this segment, and this region of the MYC TAD has the most stable secondary structural elements, even in PBS (S4A Fig). Therefore, CD measurements of MYC 120-161-TRRAP 2033–2088 were taken with increasing TFE concentrations from 10%-90% (v/v) (S4B Fig). These measurements show that, in the presence of 0–20% TFE, the resulting complex is too unstructured to warrant further measurements. However, the complex showed highly α-helical character in the presence of 20–30% TFE. There were minimal gains in secondary structure upon increasing the TFE concentration beyond 30%.

Before HSQC measurements were carried out, simple one-dimensional ^1^H-NMR spectra were collected to confirm that MYC 120–161 had a measurable W135 signal in the presence of 30% TFE-d_2_. As shown in S4C Fig, a peak in the chemical shift (~9.8 ppm) consistent with that of a tryptophan residue side-chain appears only in the presence of TFE. Additionally, the dispersion of peaks between 6–10 ppm in TFE compared to PBS demonstrates that MYC 120–161 transitions from an unfolded to a folded state.

**Fig 5:**
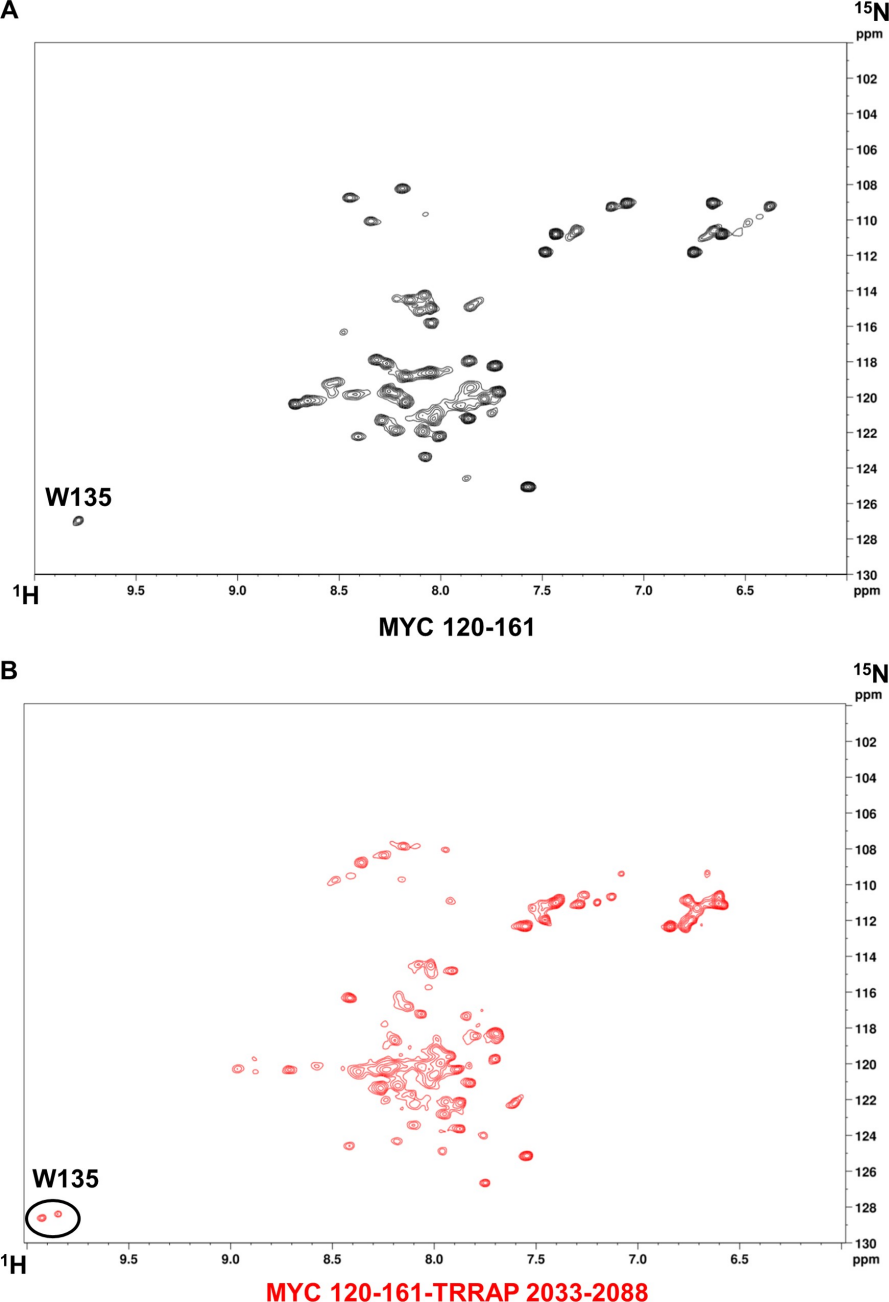
The environment of W135 in MYC 120–161 vs MYC 120-161-TRRAP 2033–2088. (A-B) ^1^H, ^15^N-HSQC spectra of MYC 120–161 (black) and MYC 120-161-TRRAP 2033–2088 (red) in 30% TFE-d2. The peak shifts of W135 in the MYC-TRRAP fusion spectrum is indicative of a binding event. The splitting of the peak suggests two stable conformations: bound and unbound states.

Next, HSQC measurements of ^15^N MYC 120–161 and MYC 120-161-TRRAP 2033–2088 were compared to perform the assignment of W135 and determine if a binding event can occur (Fig 5). In the MYC alone construct containing 54 residues, 65 peaks were resolved using NMRFAM-SPARKY’s automated peak picking (APES) utility [47]. It contained 1 P, 1 R, 2 Ns, 3 Qs, 1 W, and no H residues. The 5 Ns and Qs side-chain N-H pairs match exactly to the predicted peaks in the 108–112 ppm ^15^N and 6–7.5 ppm ^1^H region. These peaks are doublet proton peaks with the same nitrogen chemical shift. The W135 N-H side-chain pair has a nitrogen chemical shift of ~127 ppm and a hydrogen chemical shift of ~9.8 ppm, typical of a N-H indole pair.

In the 127 residue MYC-TRRAP construct, 122 peaks were resolved as above. However, this construct contained 3 Ps, 6 Rs, 4 Ns, 5 Qs, 1 W, and no H residues. More importantly, the W135 N-H side-chain pair had a split peak resonance, suggesting that it resides in two different environments and hence there are likely two different conformations for the construct. Considering both spectra, one of the two chemical shifts of the split peak can represent each of the two conformations. Since TRRAP binding requires W135 (S1B Fig), these two conformations provide evidence for the existence of a bound and an unbound MYC:TRRAP state in TFE. The ratio of intensities of these two peaks is an indicator of the ratio of bound and unbound complexes. These measurements validated the previous data and confirm that a stable 3D structure of MYC:TRRAP can exist in the presence of TFE. The assignment of W135 will be extremely useful in ligand screening assays to confirm direct binding to MYC and to observe specific disruption of the W135 chemical environment.

## Discussion

Exploiting cancer dependencies for medicinal purposes has already led to the development of mechanism-based targeted therapies. Rather than interfering with all rapidly dividing cells (chemotherapy), targeted therapy specifically blocks the growth of cancer cells by interfering with pathways needed for carcinogenesis. Numerous studies have shown that MYC is unique and essential for tumorigenesis and disease progression, and therefore, a good candidate for targeted inhibition [1]. TRRAP is a MYC MB2 cofactor and therefore therapeutically targeting MB2 will involve its interaction with TRRAP. *In vivo* studies demonstrate the therapeutic effects of MYC inhibition via the DNA-binding domain [20]. However, an MB2 deletion mutant of MYC shows drastically reduced carcinogenesis and disease progression in animal models [24]. A new generation of compounds that target MB2 will have tremendous clinical applications.

MYC is an intrinsically disordered protein and the MYC TAD is one of its IDRs. As shown here, the region of TRRAP interaction with MYC is also disordered. However, these regions of interaction acquire a structurally-stable conformation in the presence of each other. *In vitro*, the MYC:TRRAP interaction requires stabilization with a covalent linker and the presence of EG or TFE to recapitulate the binding characteristics of the interaction in cells. Co-IP experiments with EG and NMR experiments with TFE confirm that not all MYC-TRRAP fusion protein molecules form intramolecular MYC:TRRAP complexes. Nonetheless, a significant increase in complex formation is observed in both cases. This increase is MB2 dependent but does not necessarily imply that all molecules will be observed in a bound state. HSQC experiments of MYC in TFE show that W135 has a single chemical environment in MYC and two distinct environments in a MYC-TRRAP fusion. This suggests two major conformations for the MYC-TRRAP fusion protein: a bound and an unbound state.

Kosmotropic solutions can induce artificial structures and interactions. However, TFE at 30% (v/v) has been successfully used as an aid in the formation of a stable conformation for structural determination [48]. More recently, 30% (v/v) TFE has been shown to recover bioactive proteins from bacterial inclusion bodies with higher efficiency than more commonly used refolding strategies involving denaturants such as urea or guanidine [49]. Strategies for structural stabilization with TFE have been more successful when a protein has a known or predicted primarily α-helical secondary structure [50,51]. For MYC and TRRAP, both proteins have primarily predicted α-helical secondary structures within their most critical regions for the interaction (S5A Fig). In fact, a tertiary structure prediction of MYC 120–161 depicts two interacting helices where the core of MB2 (DCMW) is presented as an exposed loop between them, which is our hypothesis of how the MYC:TRRAP interaction occurs (S5B Fig). It is also important to point out that the secondary structure induced by these agents like TFE and EG on the MYC-TRRAP fusion protein are dependent on the most critical region for the interaction, i.e. MB2 (Figs 4 and 5). This suggests that the intramolecular complexes being formed by the fusion protein are dependent on the native region of interaction of the complex in cells, which gives evidence that this model system replicates the native state of the complex.

The ratio of intensities between the two peaks of W135 in the MYC-TRRAP fusion protein in an HSQC experiment can be directly correlated with the number of complexes in each of the two states described above. While NMR peak intensities can be used to evaluate compounds for their ability to disrupt the MYC:TRRAP interaction given that any change in the chemical environment of W135 can be measured and quantified using the ratio of peak intensities between the bound and unbound states, other more high-throughput compound screening assays can be developed that evaluate MYC:TRRAP inhibitors. For example, using the intrinsic fluorescence of W135, disruption by small-molecules of intramolecular complexes of the fusion MYC-TRRAP complex can be measured, resulting in the identification of possible inhibitors of the MYC:TRRAP interaction.

Finally, it may be difficult to solve the structure of the MYC:TRRAP complex in solution using this approach given the heterogeneity of the molecules present since the measurements would have to be assigned to either conformation. Protein crystallization for the purpose of X-ray diffraction would also require uniformity to form a crystal lattice.

Furthermore, it is possible that there are other factors that are required to achieve the MYC:TRRRAP complex in cells. The MYC:TRRAP complex requires more than just the most essential regions that we identified here and there are secondary sites that stabilize the complex. However, working with the smallest possible regions of interaction of each protein allows the targeting of any drug discovery approach to disrupt MB2 specifically and not any other secondary site of interaction. The use of these non-physiologic facilitators for *in vitro* experiments is justified for the purpose of evaluating such minimal domains of interaction.

Tumors regress in animal models that mimic MYC inhibition or downregulation, even those driven by other oncogenes or the lack of tumor suppressor genes [20,52–54]. However, all these models have either downregulated the MYC protein or inhibited its function by targeting the DNA-binding domain. Our data suggest that the MYC:TRRAP interaction could have a lower free energy of association, and therefore is a more desirable target for inhibition of MYC function in cancer by small-molecules than the MYC:MAX interphase. The smaller 17 amino acid MB2 region required for the MYC:TRRAP interaction could easily be disrupted by a small-molecule, which has proven extremely difficult to do for the MYC:MAX interaction because it involves the entire MYC DNA-binding domain. Only one report has studied MYC tumorigenesis in animal models deleting MB2 [24]. They tested the effects of introducing either MYC or MYC ΔMB2 in a murine isogenic model of triple-negative breast cancer derived from MCF10A cells. These observations provide the first *in vivo* evidence of an essential function of MB2 for MYC-driven tumorigenesis. Inhibiting MYC by targeting MB2 and its interaction with TRRAP could be a fruitful strategy.

The site of TRRAP interaction with MYC in its HEAT domain is an IDR. Although the structure of Tra1p (yeast homologue) has been described alone and in complex with part of NuA4 [40,55], the parallel region that interacts with MYC in humans was not resolved. The structure of Tra1p is composed of α-helical solenoid repeats, spanning both HEAT and FAT domains, which account for 86% of its mass. Furthermore, the C-terminal region of other PIKK family members show striking resemblance. The HEAT domain of TRRAP/Tra1p share remarkable similarities to DNA-PKcs, though not to the other members of the PIKK family [40,56]. HEAT repeats have been reported to critically regulate PIKK function because they are essential for DNA and protein-protein interactions that regulate activity and cellular localization [26]. The massive HEAT domain of DNA-PKcs may be part of an allosteric mechanism of modulation for DNA double-strand break repair by accommodating interactions with DNA and DNA repair factors [57]. For TRRAP, the MYC-interacting region contains the two major phosphorylation sites in TRRAP as well as a nuclear localization signal [58–60]. As is the case for DNA-PKcs, it is possible this TRRAP IDR is involved in an allosteric mechanism of modulation of TRRAP-containing HAT complexes. MYC binding could induce conformational changes to these complexes that regulate their function. GCN5 and Tip60 can acetylate histone tails *in vitro* but cannot acetylate an assembled nucleosome, presumably because they require other members of STAGA and NuA4 complexes respectively [61,62]. The only shared subunit of these complexes is TRRAP [63]. Given these observations and the similarity of the TRRAP HEAT domain structural with that of DNA-PKcs, TRRAP could be required for efficient HAT activity because it enables the presentation of lysine tails by denaturing nucleosomes. Its HEAT domain could help stabilize relaxed DNA within its large solvent-accessible channels. This model provides rationale for the essential role of TRRAP in MYC driven cancers.

## Supporting information

S1 Fig Endogenous co-IP confirmation. (A) MYC 1–190 and MYC 1–190 ΔMB2 were cloned into a CMV-PYO expression vector and transfected into HEK293T cells, then MYC was IPed with anti-PYO beads. Co-IP of endogenous TRRAP was evaluated by western blot. Endogenous TRRAP can co-IP with MYC 1–190 but requires MB2. (B) MYC, MYC ΔMB2, and MYC W135G were cloned into a CMV-PYO expression vector and transfected into HEK293T cells, then MYC was IPed with anti-PYO beads. Co-IP of endogenous TRRAP was evaluated by western blot. Endogenous TRRAP can co-IP with MYC and requires MB2 and W135.
(TIF)

S2 Fig Protein purification strategy. (A) The general protein purification strategy involved the production of a protein construct in *E*. *coli* expressed by a modified pGEX vector containing both an N-terminal GST tag and a C-terminal TS tag. (B) A Coomassie-stained SDS-PAGE after production and lysis, cleared lysates were subjected to a glutathione column and the protein construct was eluted. It was then loaded on a StrepTactin® XT column and eluted a second time with biotin. The eluate was then subjected to a cleavage reaction by TEV protease carried out at 4°C for 16h. Next, both the GST tag and TEV protease were removed on agarose-glutathione beads. The TS tag was subsequently removed on StrepTactin® XT beads. Finally, the sample was loaded on an SEC column. After this final purification step, it was concentrated, flash frozen, and stored at -80°C.
(TIF)

S3 Fig The effects of additives on MYC and TRRAP. (A-K) CD spectra of MYC 1–190, MYC 1–190 mixed with TRRAP 2033–2088, and TRRAP 2033–2088 with the indicated additives at the indicated concentration.
(TIF)

S4 Fig Endogenous co-IP confirmation. (A) CD spectra of MYC 1–190, MYC 1–190 ΔMB2, MYC 120–161, and TRRAP 2033–2088 demonstrate that all four are intrinsically disordered. The lack of significant minima at wavelengths 208 nm, 215 nm, and 222 nm suggest that these constructs lack ordered secondary structure. This is confirmed also by the overall shapes of the curves with minima at 202 nm. However, the slight minima observed at 222 nm in MYC 1–190 and MYC 120–161 suggest that there might be some α-helical structural elements present. (B) CD spectra of MYC 120-161-TRRAP 2033–2088 in 0%-90% (v/v) TFE. Increasing TFE concentration is indicated by increasing darkness in color. TFE induces a gain in α-helical secondary structure with each increase in concentration. (C) ^1^H-NMR spectra of MYC 120–161 in 1X PBS (left) and 30% TFE-d_2_ (right). Bottom panels are enlarged from 6–10 ppm of the above spectra. The spectrum of MYC 120–161 in PBS indicates the presence of significant unstructured elements based on the large cluster of severely overlapped peaks. However, in the presence of TFE, the peaks become well-dispersed and individual peaks can be distinguished, which indicates a well-folded protein.
(TIF)

S5 Fig Structure predictions MYC 120–161 and TRRAP 2033–2088. (A) JPred 4 [64] secondary structure predictions of MYC 120–161 and TRRAP 2033–2088. Both are predicted to contain alpha-helical elements present. (B) Models of structure predictions using NMRFAM Ponderosa Prediction Server (POND-PRED) [42] depicting possible conformational states of MYC 120–161. D132, C133, M134, and W135 are shown in red.
(TIF)

S1 Raw Images Raw images for all gels and blots used in figures. (PDF)
