# Supplementary figures and images for: Formation of a structurally-stable conformation by the intrinsically disordered MYC:TRRAP complex
Source: PLoS One. 2019 Dec 2;14(12):e0225784. doi: 10.1371/journal.pone.0225784 (PMC6886782; doi:10.1371/journal.pone.0225784)

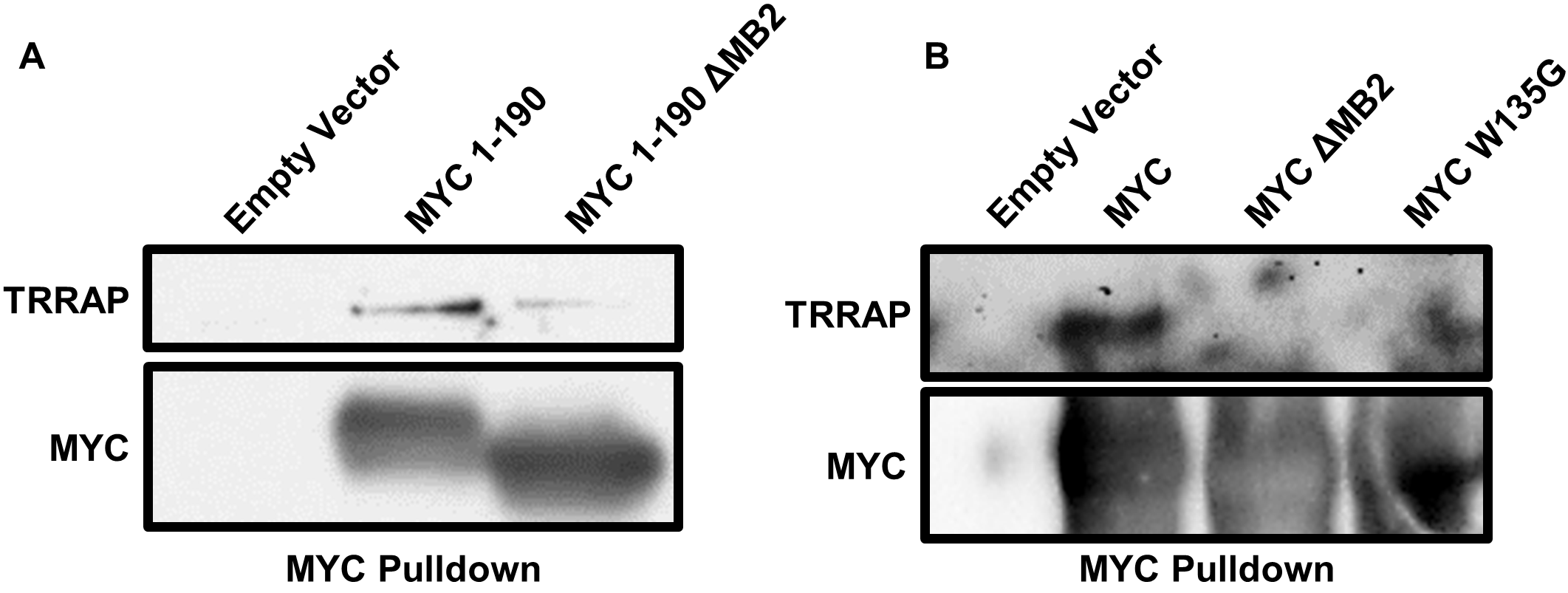

Supplement: S1 Fig — (A) MYC 1–190 and MYC 1–190 ΔMB2 were cloned into a CMV-PYO expression vector and transfected into HEK293T cells, then MYC was IPed with anti-PYO beads. Co-IP of endogenous TRRAP was evaluated by western blot. Endogenous TRRAP can co-IP with MYC 1–190 but requires MB2. (B) MYC, MYC ΔMB2, and MYC W135G were cloned into a CMV-PYO expression vector and transfected into HEK293T cells, then MYC was IPed with anti-PYO beads. Co-IP of endogenous TRRAP was evaluated by western blot. Endogenous TRRAP can co-IP with MYC and requires MB2 and W135. (TIF) [file pone.0225784.s001.tif]

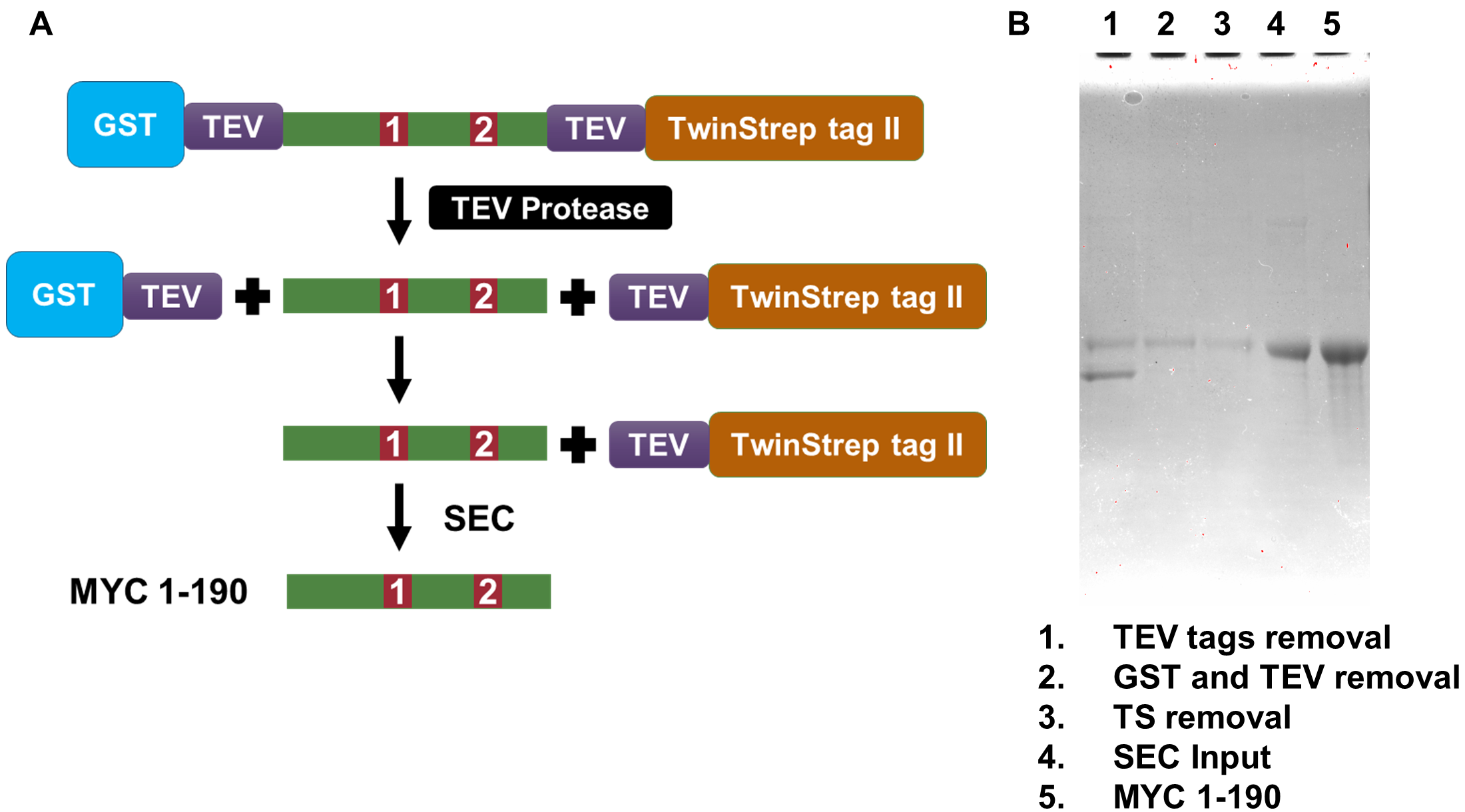

Supplement: S2 Fig — (A) The general protein purification strategy involved the production of a protein construct in E. coli expressed by a modified pGEX vector containing both an N-terminal GST tag and a C-terminal TS tag. (B) A Coomassie-stained SDS-PAGE after production and lysis, cleared lysates were subjected to a glutathione column and the protein construct was eluted. It was then loaded on a StrepTactin® XT column and eluted a second time with biotin. The eluate was then subjected to a cleavage reaction by TEV protease carried out at 4°C for 16h. Next, both the GST tag and TEV protease were removed on agarose-glutathione beads. The TS tag was subsequently removed on StrepTactin® XT beads. Finally, the sample was loaded on an SEC column. After this final purification step, it was concentrated, flash frozen, and stored at -80°C. (TIF) [file pone.0225784.s002.tif]

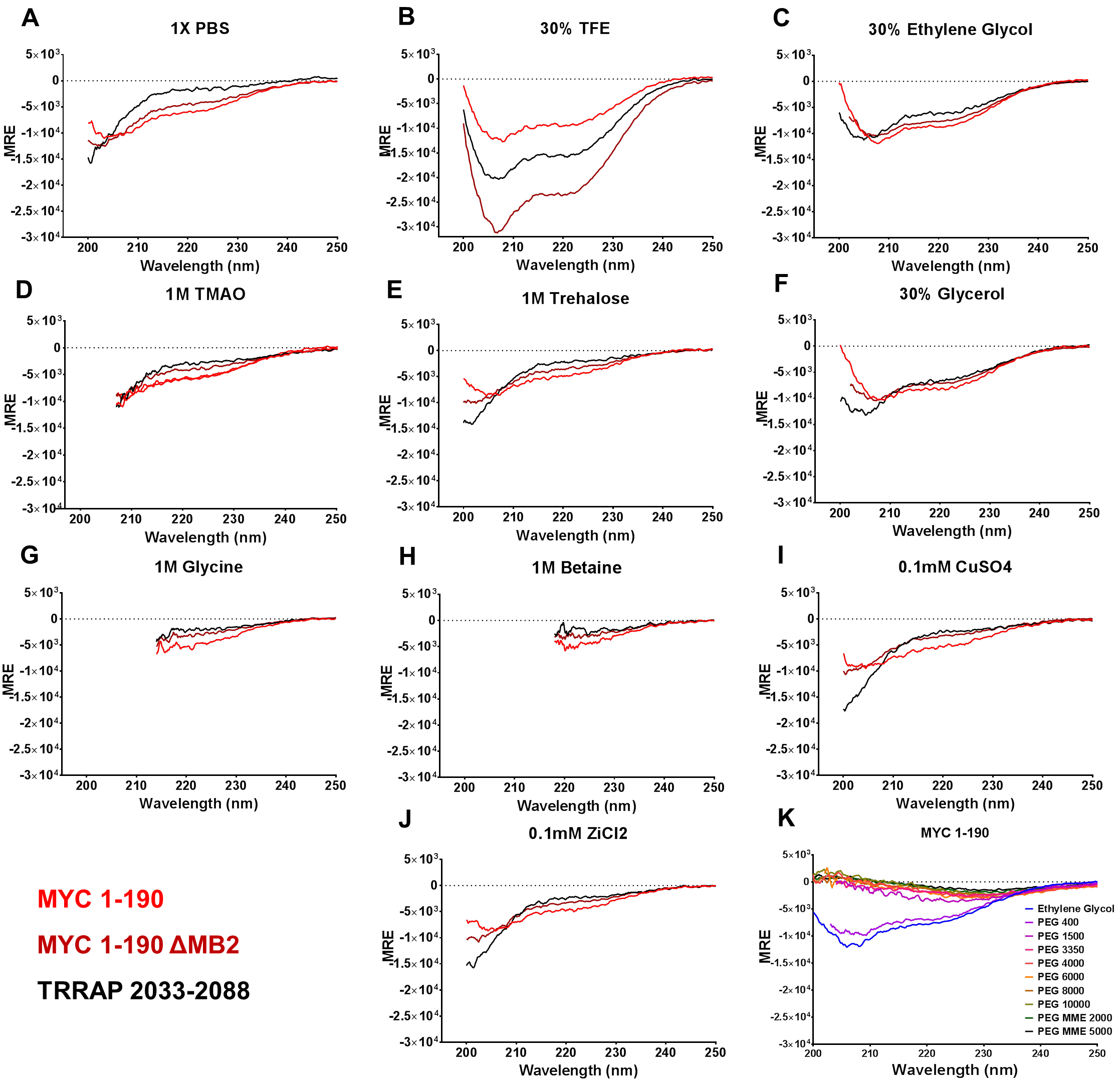

Supplement: S3 Fig — (A-K) CD spectra of MYC 1–190, MYC 1–190 mixed with TRRAP 2033–2088, and TRRAP 2033–2088 with the indicated additives at the indicated concentration. (TIF) [file pone.0225784.s003.tif]

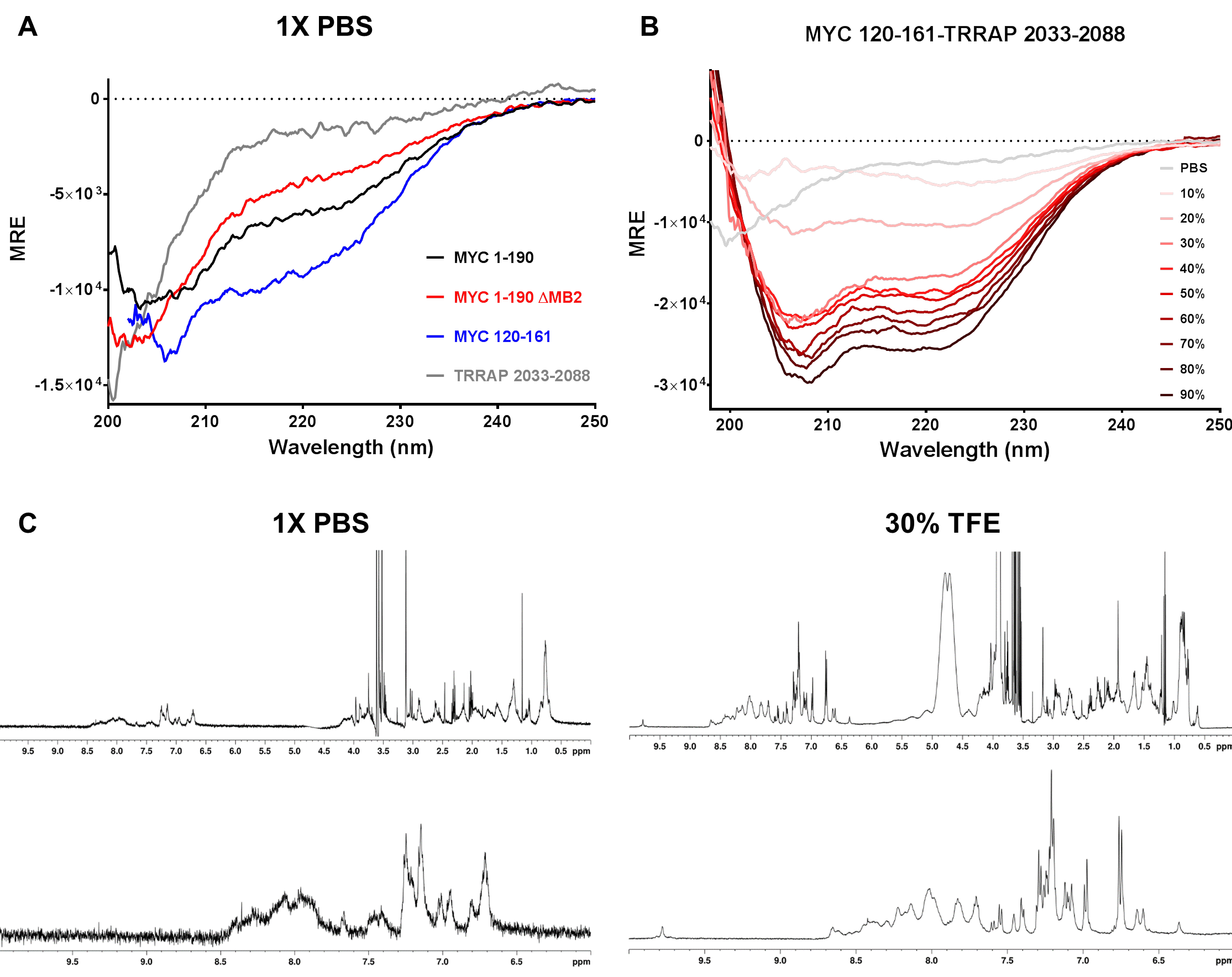

Supplement: S4 Fig — (A) CD spectra of MYC 1–190, MYC 1–190 ΔMB2, MYC 120–161, and TRRAP 2033–2088 demonstrate that all four are intrinsically disordered. The lack of significant minima at wavelengths 208 nm, 215 nm, and 222 nm suggest that these constructs lack ordered secondary structure. This is confirmed also by the overall shapes of the curves with minima at 202 nm. However, the slight minima observed at 222 nm in MYC 1–190 and MYC 120–161 suggest that there might be some α-helical structural elements present. (B) CD spectra of MYC 120-161-TRRAP 2033–2088 in 0%-90% (v/v) TFE. Increasing TFE concentration is indicated by increasing darkness in color. TFE induces a gain in α-helical secondary structure with each increase in concentration. (C) 1H-NMR spectra of MYC 120–161 in 1X PBS (left) and 30% TFE-d2 (right). Bottom panels are enlarged from 6–10 ppm of the above spectra. The spectrum of MYC 120–161 in PBS indicates the presence of significant unstructured elements based on the large cluster of severely overlapped peaks. However, in the presence of TFE, the peaks become well-dispersed and individual peaks can be distinguished, which indicates a well-folded protein. (TIF) [file pone.0225784.s004.tif]

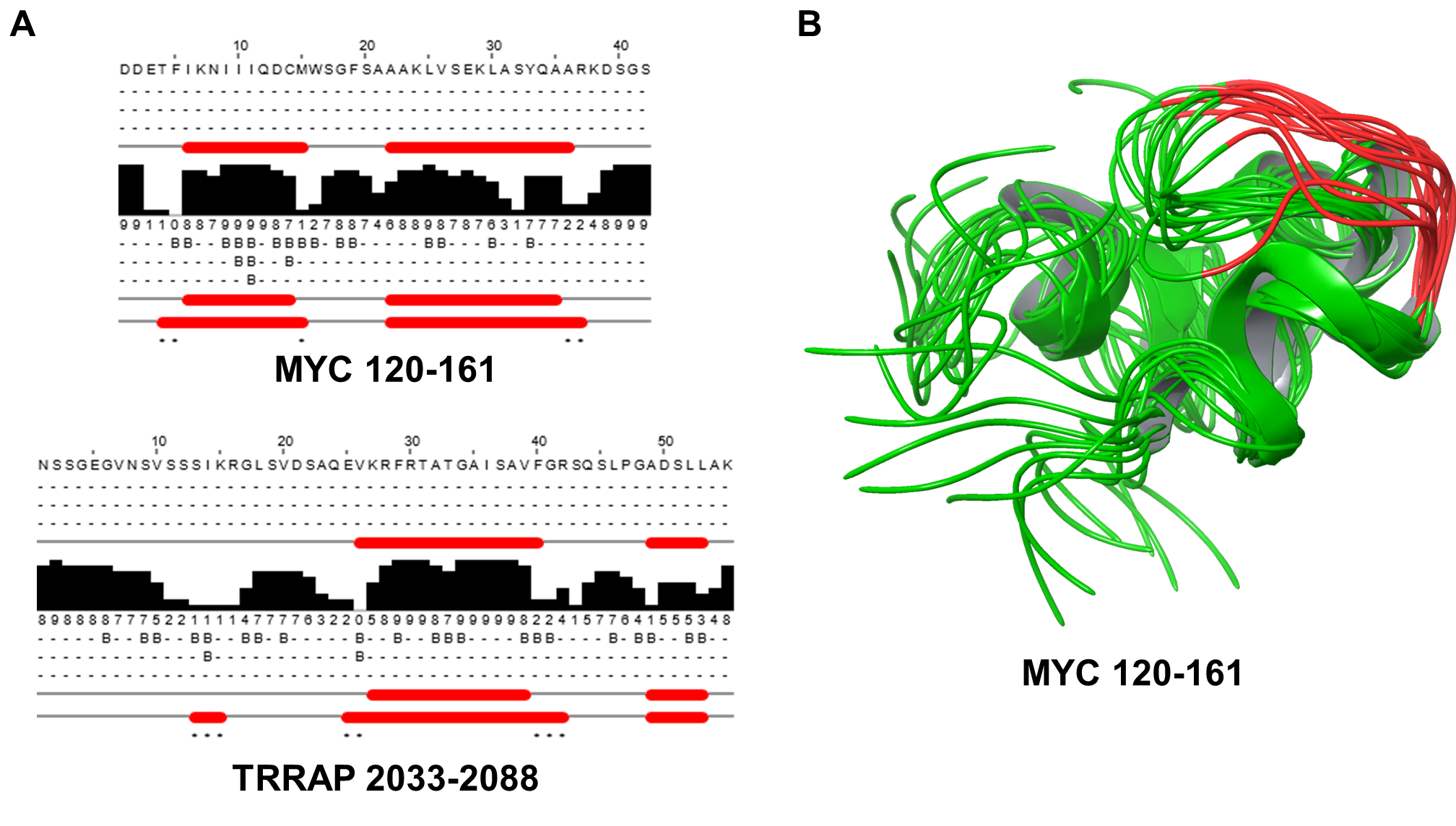

Supplement: S5 Fig — (A) JPred 4 [64] secondary structure predictions of MYC 120–161 and TRRAP 2033–2088. Both are predicted to contain alpha-helical elements present. (B) Models of structure predictions using NMRFAM Ponderosa Prediction Server (POND-PRED) [42] depicting possible conformational states of MYC 120–161. D132, C133, M134, and W135 are shown in red. (TIF) [file pone.0225784.s005.tif]
