# Supplementary material for: Formation of a structurally-stable conformation by the intrinsically disordered MYC:TRRAP complex
Source: PLoS One. 2019 Dec 2;14(12):e0225784. doi: 10.1371/journal.pone.0225784 (PMC6886782; doi:10.1371/journal.pone.0225784)
Supplement: S1 Raw Images — (PDF) [file pone.0225784.s006.pdf]

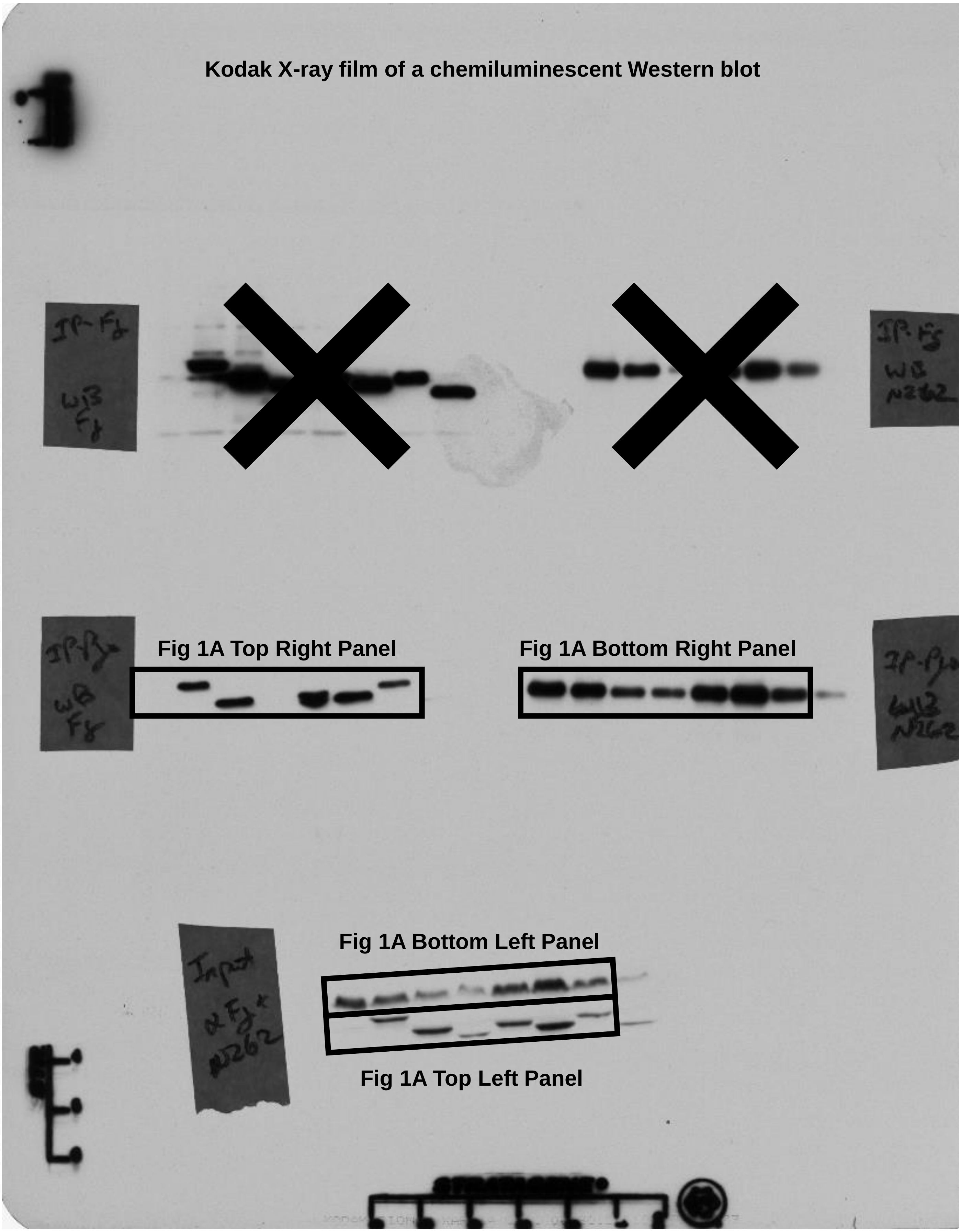
Kodak X-ray film of a chemiluminescent Western blot
Fig 1A Top Right Panel
Fig 1A Bottom Right Panel
Fig 1A Bottom Left Panel
Fig 1A Top Left Panel

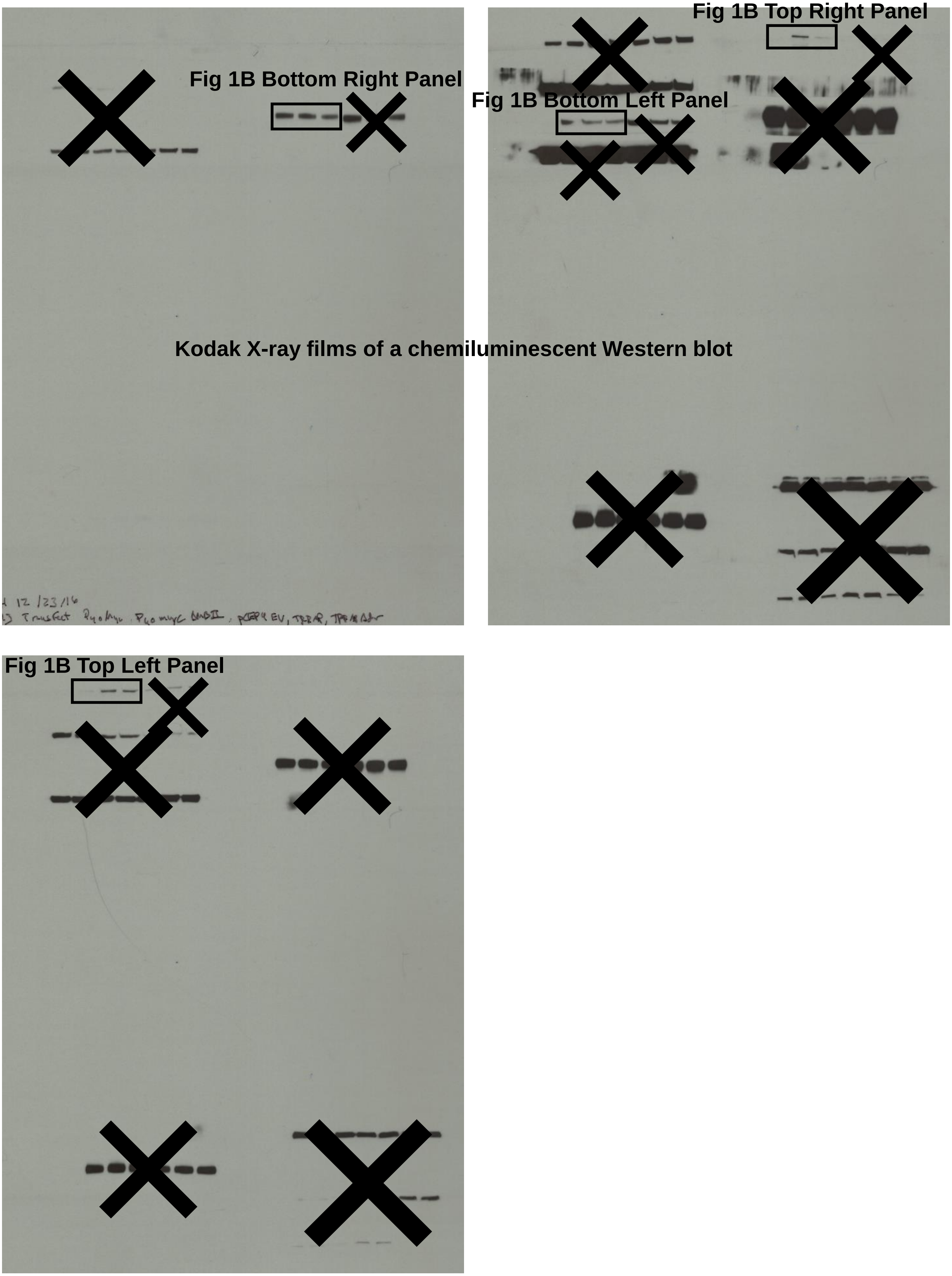

Kodak X-ray films of a chemiluminescent Western blot

Kodak X-ray film of a chemiluminescent Western blot

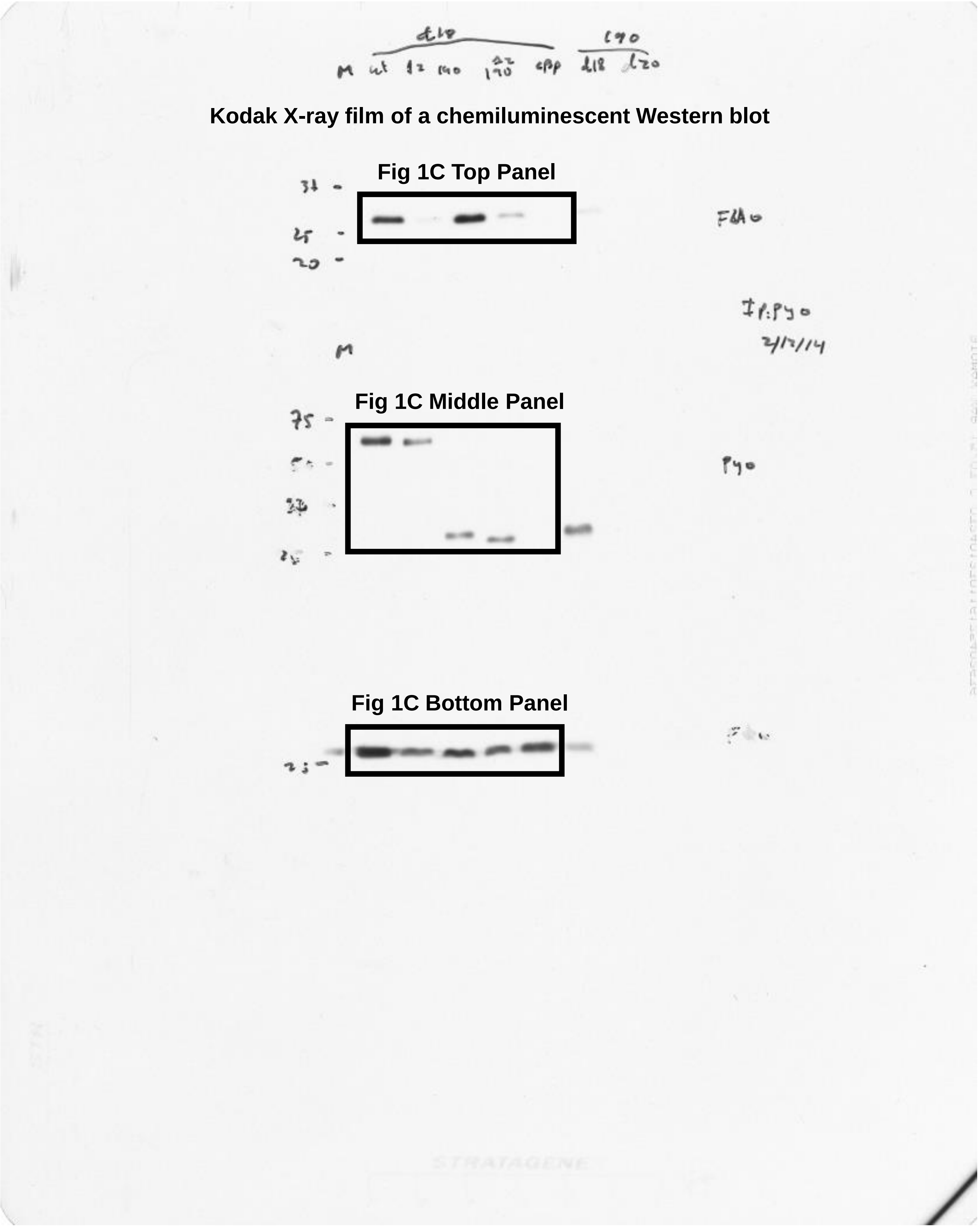

LI-COR Odyssey scan of a Coomassie-stained SDS-PAGE

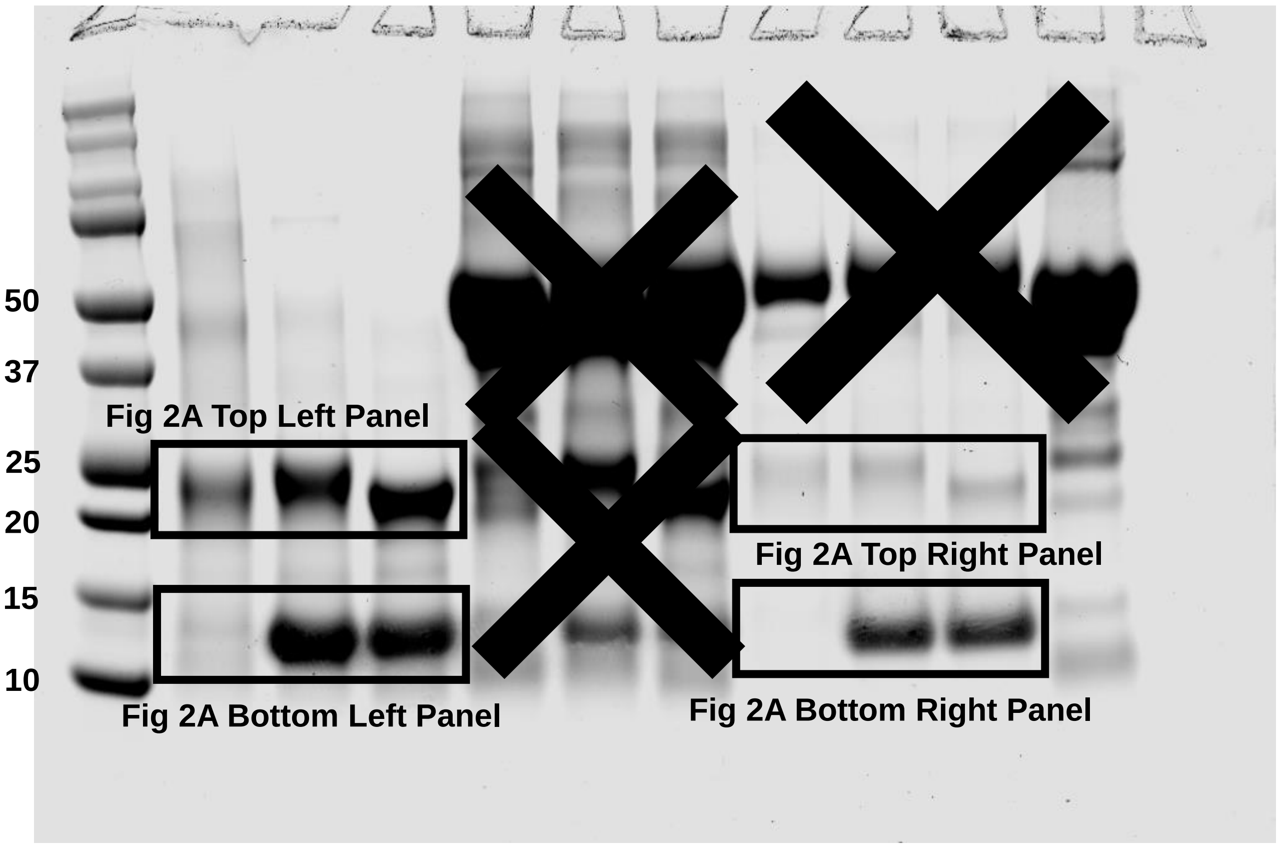

Bio-Rad ChemiDoc Molecular Imager scan of a chemiluminescent Western blot

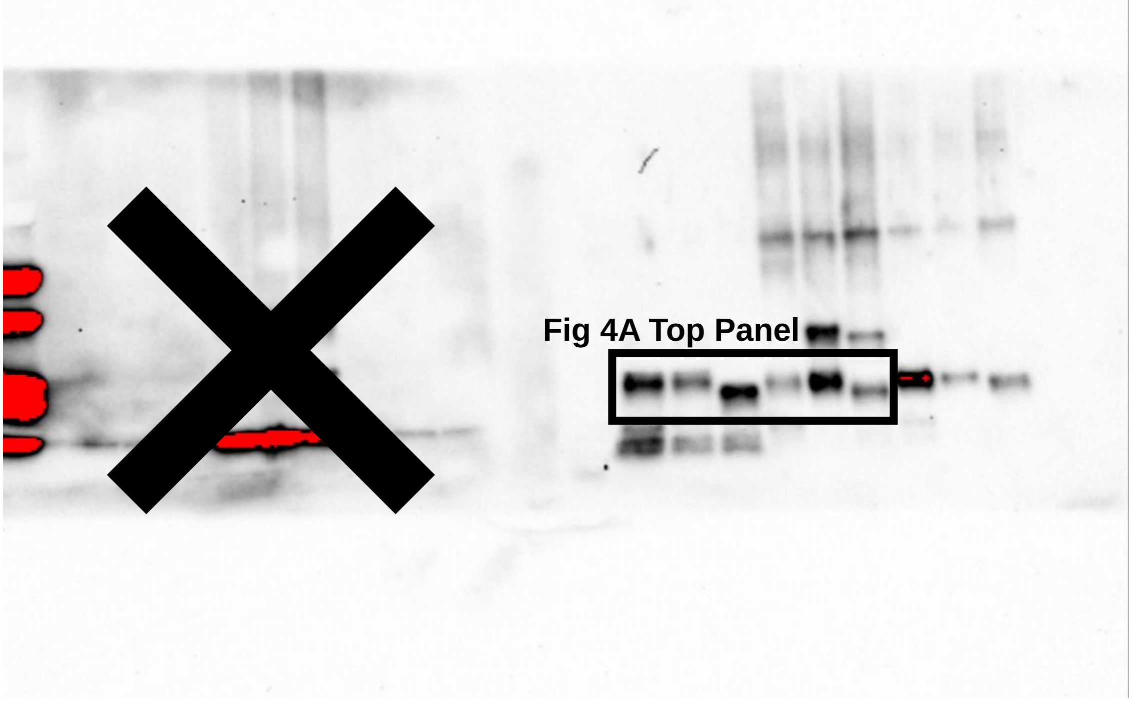

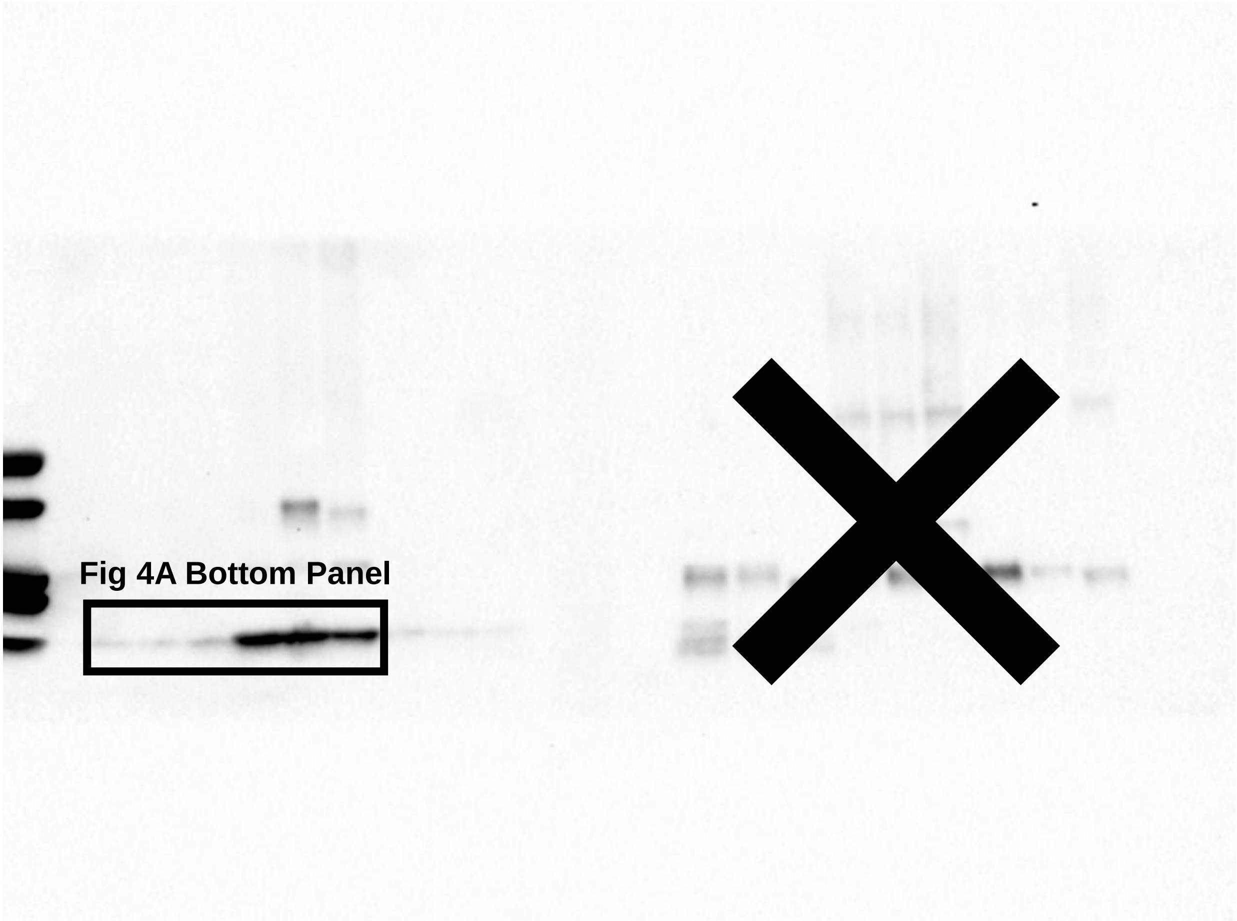

Kodak X-ray film of a chemiluminescent Western blot

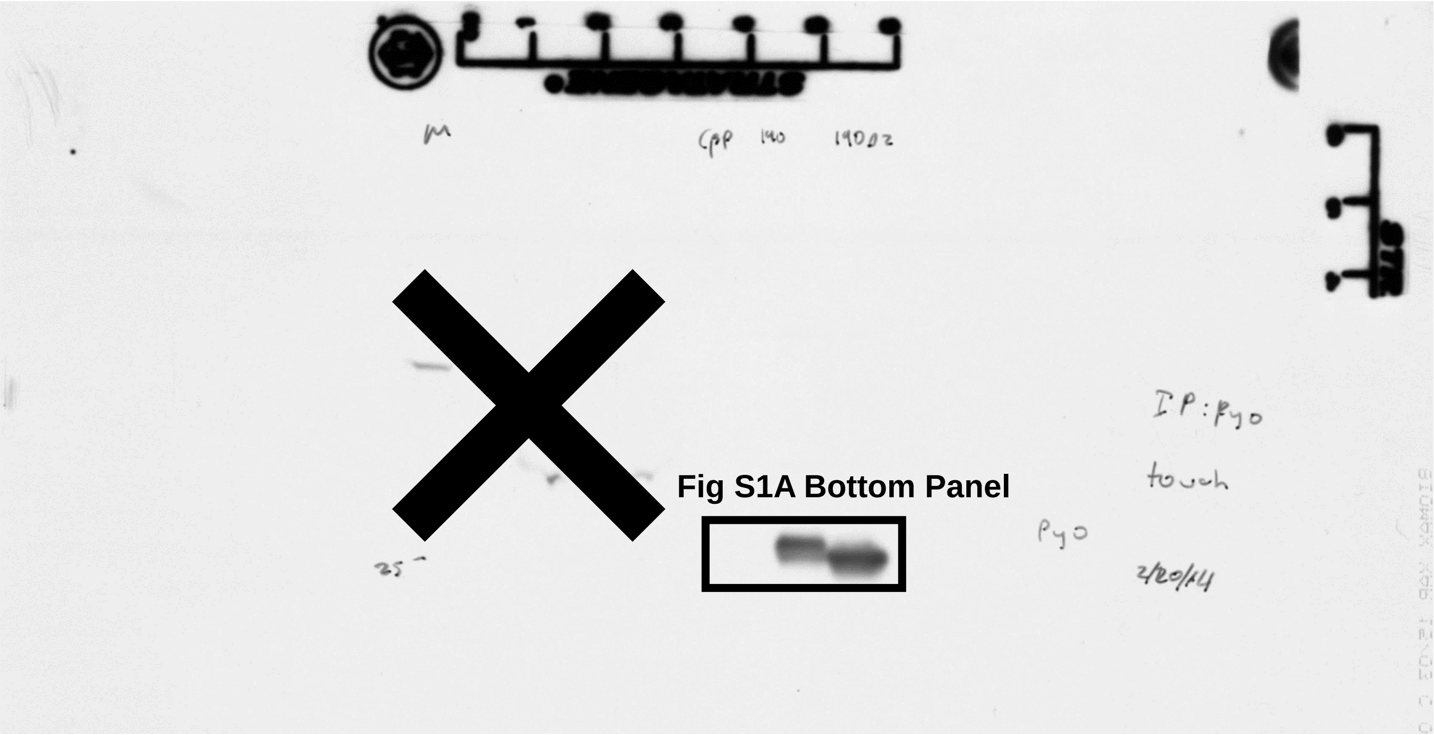

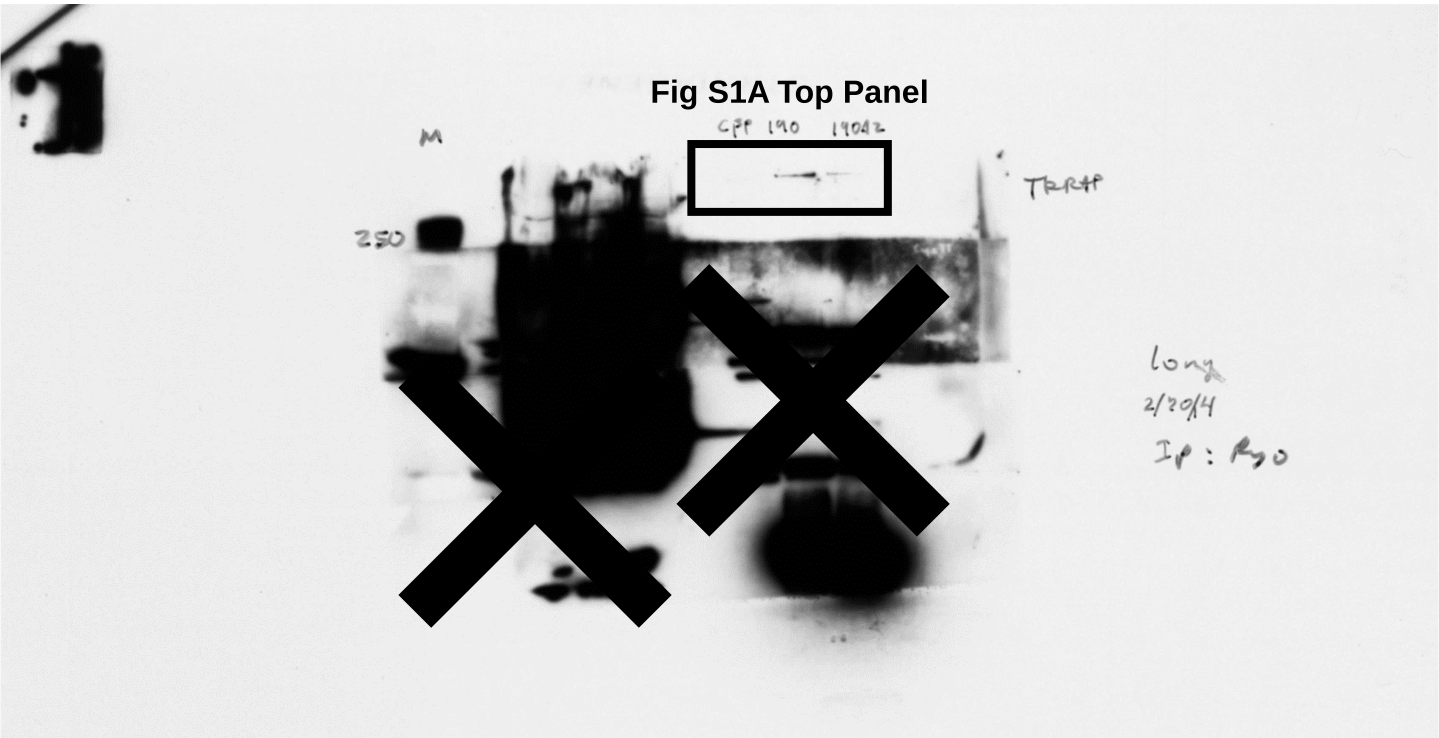

Bio-Rad ChemiDoc Molecular Imager scan of a chemiluminescent Western blot

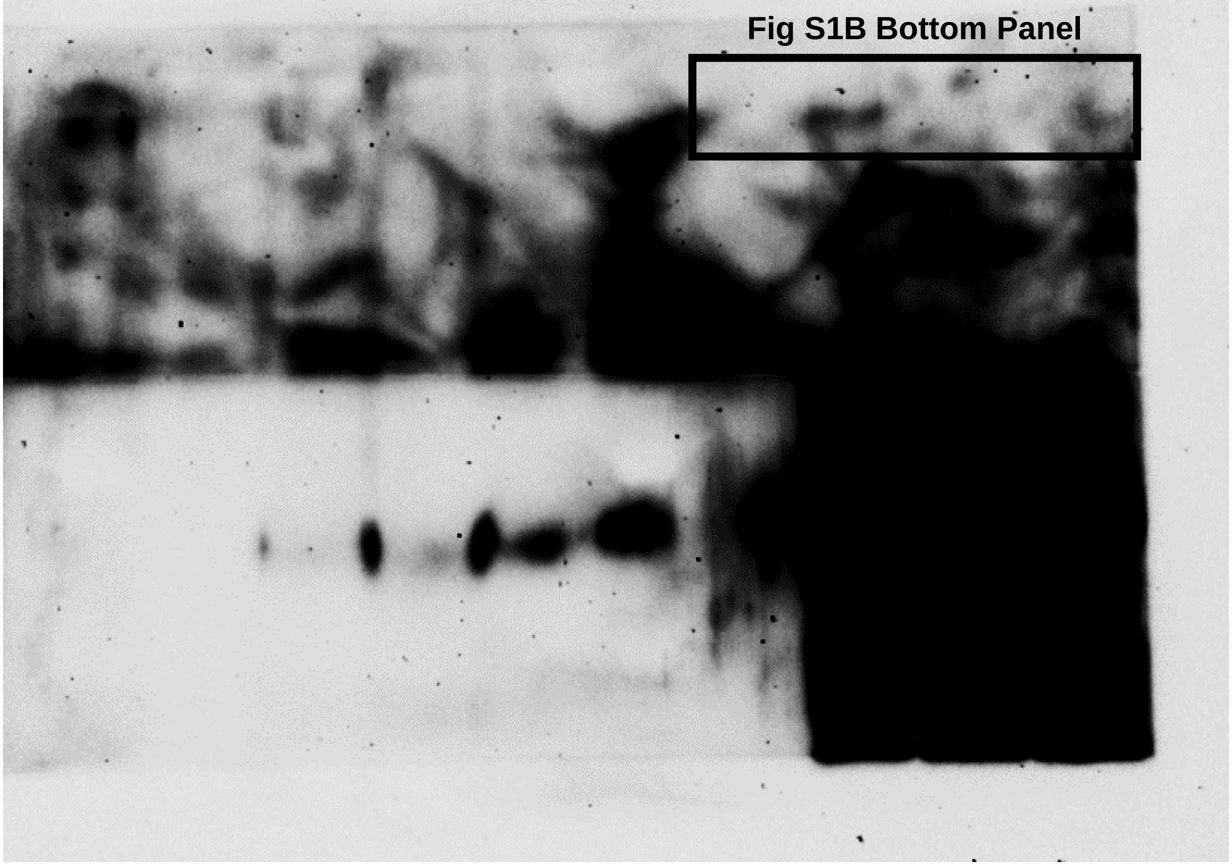

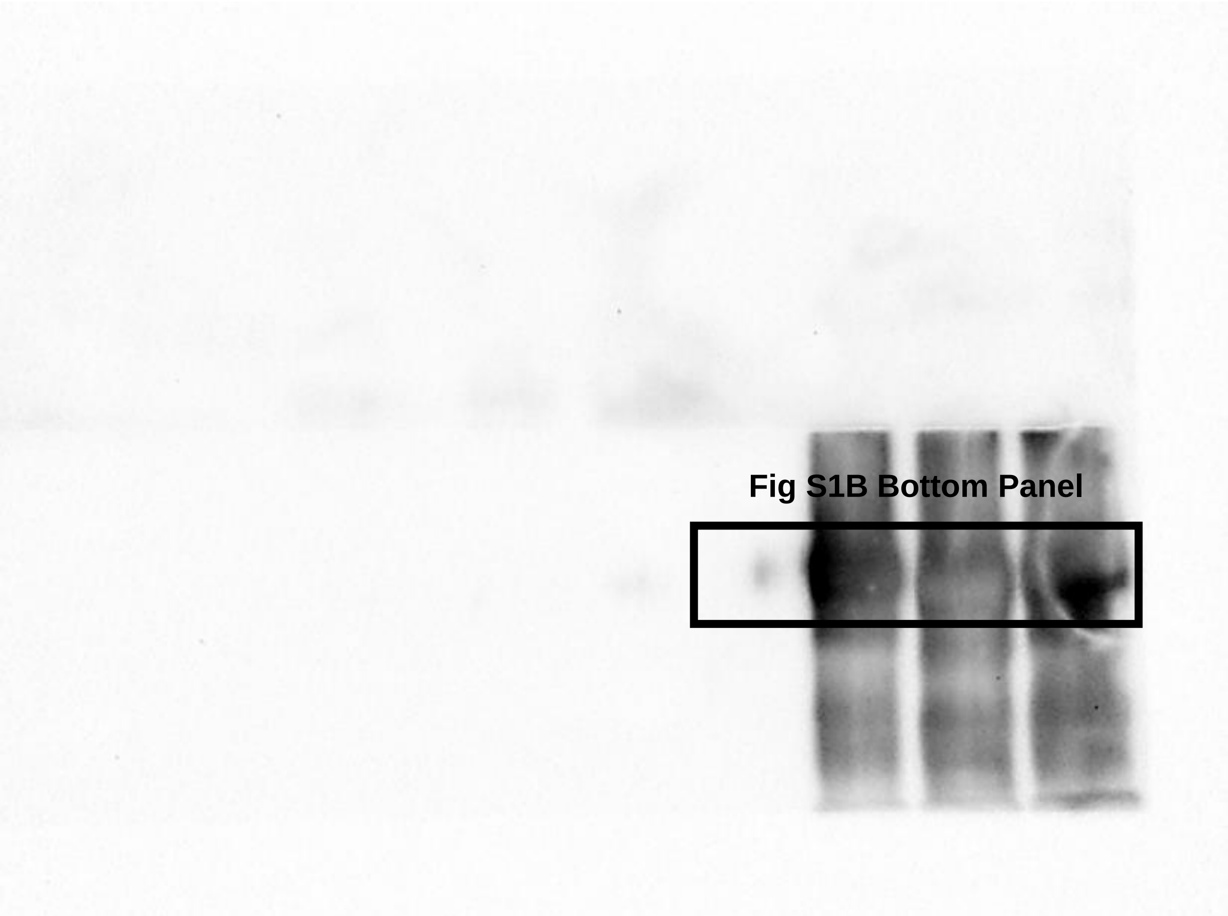

Bio-Rad ChemiDoc Molecular Imager scan of a Coomassie-stained SDS-PAGE

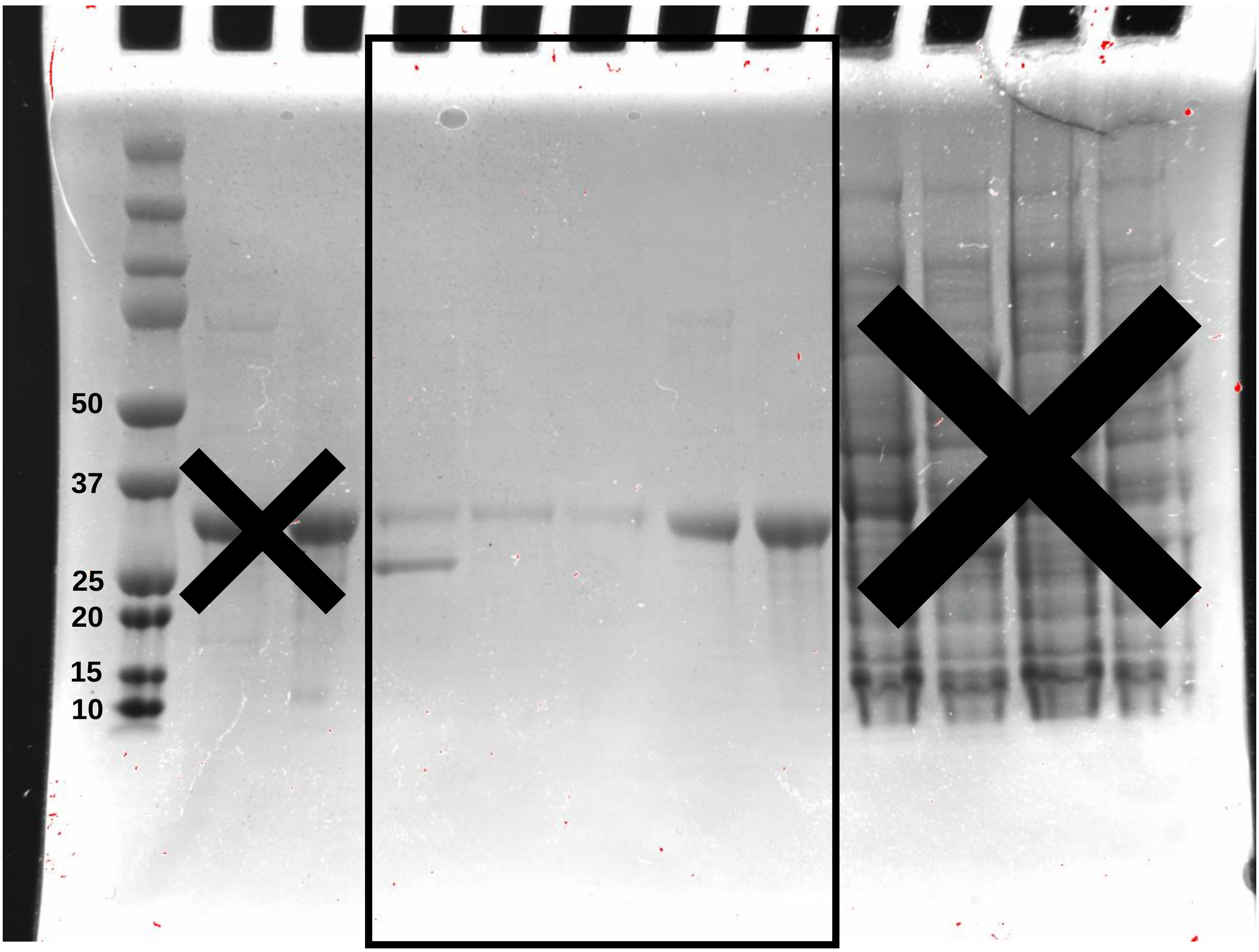

Fig S2B Panel
